# Immuno Affinity SELEX for Simple, Rapid, and Cost-Effective Aptamer Enrichment and Identification against Aflatoxin B1

**DOI:** 10.3389/fmicb.2016.01909

**Published:** 2016-12-01

**Authors:** Keerthana Setlem, Bhairab Mondal, Shylaja Ramlal, Joseph Kingston

**Affiliations:** Microbiology Division, Defence Food Research LaboratoryMysore, India

**Keywords:** aflatoxin B1, immunoaffinity SELEX, ELONA, G score, spiking study

## Abstract

Aflatoxins are naturally occurring mycotoxins that contaminate food and agro commodities, leading to acute and chronic health conditions in human and animals. In the present work, an attempt was made to generate high-affinity single stranded DNA aptamers that specifically bind to Aflatoxin B1 (AFB1) by a modified Systemic Evolution of Ligands by Exponential Enrichment (SELEX) procedure with the aid of Immunoaffinity columns. Ten rounds of SELEX and alternating three counter SELEX rounds with a cocktail of related and other mycotoxins were performed to enhance the specificity. Resultant 105 aptamers were clustered into 12 groups according to their primary sequence homology. Candidates with lowest Gibbs free energy (dG value) and unique stem loop structures were selected for further characterization. Aptamers, AFLA5, AFLA53, and AFLA71 exhibiting lower *K*_d_ values (50.45 ± 11.06, 48.29 ± 9.45, and 85.02 ± 25.74 nM) were chosen for development of ELONA and determination of purification ability of toxin. The detection limit (LOD) of AFLA5 and AFLA71 was 20 and 40 ng/ml, respectively. HPLC analysis implied that selected aptamers were able to recover and quantify 82.2 to 96.21% (LOQ – 53.74 ng) and 78.3 to 94.22% (LOQ – 66.75 ng) of AFB1 from spiked corn samples, respectively. These findings indicate, immunoaffinity based SELEX can pave an alternative approach to screen aptamers against mycotoxin detection and purification.

## Introduction

Aflatoxins are highly toxic difuranocoumarin derivatives produced by the genus *Aspergillus*, mainly by *Aspergillus flavus*, *A. parasiticus*, and *A. nomius* (Yu, 2012; Markov et al., 2013). Six out of eighteen different types of aflatoxins have been identified as the most predominant contaminants in food, agriculture, and dairy products that are designated as Aflatoxin B1, B2, G1, G2, M1, and M2 (Bakırdere et al., 2012). Among these, Aflatoxins B1 (AFB1) is classified as a group 1A carcinogen by International Agency for Research on Cancer (IARC) since it leads to liver damage and hepatocellular carcinoma apart from plentiful of deleterious health conditions (IARC, 2004; Liu and Wu, 2010; Marin et al., 2013). Therefore, aflatoxin contamination is a major concern in global food safety, urging many countries to set stringent regulations on their occurrences. The guidelines set by Food Safety and Standards Authority of India limit aflatoxins to 15 and 30 ppb in cereals and spices, respectively (FSSAI, 2015). Additionally, Food and Drug Administration (FDA) has set permissible levels to 20 ppb in food and 300 ppb in livestock feed; whereas, European Union (EU) stipulate an acceptable range upto 2 ppb of AFB1 and 4 ppb of total aflatoxins in cereal products (European Union Commission, 2006; Le et al., 2010).

Till date, various techniques like High Performance Liquid Chromatography (HPLC), Liquid Chromatography Mass Spectrometry (LC-MS), and Gas Chromatography Mass Spectrometry (GC-MS) are officially accepted as qualitative and quantitative analytical methods for AFB1 detection. These analytical methods are laborious, expensive with requirement of sophisticated equipment, skilled personnel and complicated sample preparation processes that limit their application to laboratories (Köppen et al., 2010; McDaniel et al., 2011). Molecular techniques like PCR, RT-PCR are available for the detection of aflatoxigenic fungi but matrix associated inhibitors in the samples may lead to inaccurate results (Levin, 2012). Immuno assays such as Enzyme Linked Immunosorbent Assays (ELISA), fluorescence polarization immunoassay, immuno chromatographic assay and immunosensors have paved potential path for development of rapid detection systems in the field of mycotoxins (Saha et al., 2007; Suzuki et al., 2007; Liu et al., 2008; Kanungo et al., 2011; Wang et al., 2013; Venkataramana et al., 2015). However, high production costs, instability of antibodies in different environmental conditions restrict their applications (Shim et al., 2014). In this regard, there is an increasing need for the development of suitable alternatives for detection of mycotoxin contamination in food and agro commodities.

Aptamers are ssDNA or RNA oligonucleotides widely investigated as promising alternatives for direct detection of various targets and can be generated by an *in vitro* approach known as Systematic Evolution of Ligands by Exponential Enrichment (SELEX; Ellington and Szostak, 1990; Tuerk and Gold, 1990; Stoltenburg et al., 2007). The feasibility and flexibility of aptamers in terms of small size, ease of synthesis, labeling, reproducibility, non-toxicity, and lack of immunogenicity have benefited them to evolve as ideal substitutes in target capture and detection. Additionally, aptamers are capable of retaining their reactivity under a wide range of environmental conditions (Famulok et al., 2007; Iliuk et al., 2011). Recently, various high throughput SELEX technologies have been investigated for development of aptamers against mycotoxins such as Aflatoxins (AFB1, AFB2, and AFM1), Fumonisin B1 (FB1), Zearalenone (ZEA), T2 toxin and Ochratoxin A (OTA; Le et al., 2010; McKeague et al., 2010, 2014; Chen et al., 2013, 2014; Ma et al., 2014, 2015; Malhotra et al., 2014). In most of these reports, mycotoxins are coupled to magnetic nanoparticles, sepharose, agarose based resin, or streptavidin beads prior to selection and screening of aptamers. These activation procedures involve complex chemical reactions to anchor the targets onto the matrices apart from altering their native structure and consequently reducing aptamer affinity (Chen et al., 2014).

In the present study, an attempt was made to select specific aptamers against AFB1 using immunoaffinity column (IA column) based SELEX, where related and other mycotoxins were used for negative selection. This ensured the enrichment of AFB1 specific aptamers. The representative aptamers from the obtained population were subjected to binding assays to evaluate their affinities, specificities, and relative dissociation constants. Employing highly specific aptamers, Enzyme Linked Oligonucleotide Assay (ELONA) was developed for rapid and easy detection of AFB1. Toxin purification ability of aptamers was tested in spiked corn samples and confirmed by HPLC. These observations accredited that, IA column based SELEX method can be used as an alternative approach in selection of versatile aptamers for rapid detection and purification of mycotoxins from diverse sources.

## Materials and Methods

### Materials

Initially, ssDNA library, aptamer sequences, primers, and mycotoxins used in this study were obtained from Sigma-Aldrich (India), except mentioned specifically. All the solutions were prepared with ultra high purity water from Millipore water purification system. The chemicals and solvents used in present study are listed in the Supporting Information 1.

### Synthesis of ssDNA Library

The single stranded DNA (ssDNA) library used in the present study was designed with a randomized region of 40 nucleotides flanked by two known sequences of 20 nucleotides on either side. The two known sequences served as binding regions for forward and reverse primers in PCR amplification. The nucleotide sequences of the library and the primers used in the study are listed in Supporting Table 1.

### Optimization of PCR Conditions

In order to generate ssDNA, asymmetric PCR amplification (Mondal et al., 2015) of aptamer library was carried out with each reaction consisting of 4 μl of 1X PCR buffer, 3.2 μl of 1.6 mM MgCl_2_, 2 μl of 0.2 mM dNTPs, 0.4 μl of 2U/μl of Taq DNA polymerase, 1 μl of template library, 27.4 μl of distilled water and different ratios of forward to reverse primers (1.1:0.9, 1.2:0.8, 1.3:0.7, 1.4:0.6, 1.5:0.5, 1.6:0.4, 1.7:0.3, 1.8:0.2, 1.9:0.1, and 2.0:0). The PCR was performed in thermocycler (Bio-Rad, India) with following amplification conditions: initial denaturation at 94°C for 5 min and 30 cycles of 94°C for 45 s, 56°C for 45 s, 72°C for 45 s and final extension at 72°C for 8 min. PCR amplicons were separated by 2% agarose gel electrophoresis and desired product size was purified using Qiagen MiniElute gel extraction kit following manufacturer’s protocol.

### Modified Immunoaffinity Column Based SELEX

**FIGURE 1:**
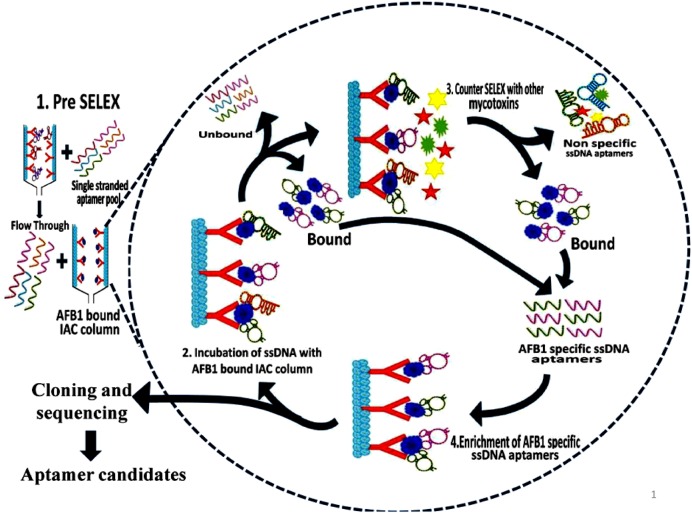
**Schematic illustration of aptamer selection procedure.** The figure depicts the SELEX procedure in the selection of aptamers against Aflatoxin B1 (AFB1) using IA columns.

Aptamers exhibiting affinity to AFB1 were selected using modified immunoaffinity based SELEX (**Figure 1**). Detailed methodology is provided in the Supporting Informations 2 and 3.

Initially, pre-SELEX was performed with 2 nmol denatured aptamer library dissolved in 500 μl of 1X binding buffer (1X BB; 5 mM MgCl_2_ in Dulbecco’s phosphate buffered saline, pH 7.4 ± 0.2) and mixture was incubated for 30 min in empty preconditioned IA column to partition aptamers having affinity to column components. The collected flowthrough was pooled and concentrated by PCR clean up kit following manufacturer’s protocol (Supporting Information 3A). The eluted DNA was used as template library for selection of aptamers against AFB1 in further rounds of SELEX. In each round of SELEX, 100 ng/μl of pre-prepared, denatured ssDNA pool was incubated with AFB1 (200 ng/ml) bound to IA column and washed with 500 μl of 1X BB (Supporting Information 3B). Bound aptamer pool was eluted with methanol. DNA from pooled elutes was recovered and dissolved in 50 μl of distilled water and concentration of obtained DNA was measured by Nanodrop-2000 (Thermo Scientific, India). The eluted DNA was amplified by earlier standardized PCR conditions and used in subsequent rounds of SELEX. The concentration and specificity of ssDNA pool toward AFB1 from each round was monitored by Nanodrop-2000 (Thermo Scientific, India) and ELONA (Mondal et al., 2015) using biotin-labeled aptamer pool (Supporting Information 4). To enhance the specificity of the aptamers, counter SELEX was introduced after 3rd, 5th, and 7th rounds. Aflatoxins (AFB2, AFG1, and AFG2) were conjugated to BSA molecule according to Cervino et al. (2008) with certain modifications and commercially procured BSA conjugated Ochratoxin A (OTA-BSA) were employed in the counter SELEX (Supporting Information 3B).

### Cloning, Sequencing, and Structural Analysis of Aflatoxin Specific Aptamers

High affinity aptamer pool from 10th round was cloned using TOPO TA cloning kit following manufacturer’s instructions. Positive clones were selected and sequenced utilizing the in-house sequencing facility (ABI 3500 Genetic Analyzer, Applied Biosystems, India) (Supporting Information 5). Obtained sequences were analyzed by using online multiple sequence alignment tool, Multalin^1^ (Corpet, 1988). Possible secondary structures and Gibbs free energies (dG) of selected aptamers were predicted using the M-fold software^2^ at 26°C in 150 mM (Na+) and 1 mM (Mg++; SantaLucia, 1998; Zuker, 2003). Putative Quadruplex forming G Rich Sequences (QGRS) of high affinity aptamers using QGRS Mapper software^3^ were predicted (Kikin et al., 2006).

### Identification of Aflatoxin B1 Specific Aptamers

Binding affinity of aptamer pool and individual aptamers were evaluated by ELONA (Supporting Figure S4; Supporting Information 4). Microtiter plates were coated with BSA conjugated AFB1 (AFB1-BSA) at a concentration of 200 ng/ml (carbonate-bicarbonate buffer, pH 9.6 ± 0.2). After blocking with BSA (3%), biotin labeled ssDNA aptamers (250 ng/μl) were added and incubated for 1 h at 37°C. The plates were washed with 1X PBST prior to incubation with Streptavidin-HRP (1:3000 dilutions) at 37°C for 45 min. After incubation, plates were washed thoroughly and color was developed using chromogenic substrate (OPD/H_2_O_2_). The reaction was stopped using H_2_SO_4_ (2N) and absorbance was measured at 490 nm using Infinite M1000 spectrophotometer (TECAN, India). For evaluation of cross reactivity, the mentioned assay was repeated with other BSA conjugated mycotoxins (AFB2, AFG1, AFG2, and OTA) that were prepared according to Cervino et al. (2008).

### Determination of Apparent Dissociation Constant (*K*_d_) of Selected Aptamers

Similar ELONA was performed for determination of apparent dissociation constants (Martín et al., 2013), where constant amounts of AFB1-BSA conjugate (200 ng/ml) was titrated with increasing concentrations (0–350 nM) of biotinylated aptamers. The resultant absorbance values were fitted in *y* = *B*_max_^∗^*x*/(*K*_d_+*x*) via non-linear regression analysis, using Graph Pad Prism 6 software. Additionally, the detection potential of high affinity binders was tested with a various concentrations of AFB1-BSA (10–250 ng/ml) against constant amounts of biotinylated aptamers (Supporting Figure S4; Supporting Information 6).

### Determination of Aflatoxin B1 in Spiked Food Samples

Purchased corn samples were surface sterilized by rinsing with water followed by rapid shaking in 1% Sodium hypochlorite + 0.1% Tween 20 for 10–15 min and final washing 3–5 times for 5 min in sterile water and were grounded mechanically. For evaluating the purifying ability of selected aptamers, AFB1 ranging from 10 to 250 ng/ml was resuspended in methanol and incubated with 5 gm of powdered corn samples for 3 h. Toxin extraction from spiked samples was carried according to Stroka et al. (2000), with certain modifications (Supporting Information 7). Obtained toxin extracts were air dried in dark to remove remnants of organic solvents and resuspended in 1X BB (500 μl) and allowed to react with biotinylated ssDNA bound onto the streptavidin coated microtiter plates and eluted with methanol: water (80:20) (**Figure 7**). The recovery of eluted AFB1 was confirmed and quantified by HPLC (JASCO, UK) analysis according to Priyanka et al. (2014) with certain modifications. The chromatographic analysis of AFB1 from naive and spiked samples was carried on RP-C18 column (3 μm, 250 mm × 46 mm) using mobile phase consisting of water: acetonitrile: acetic acid in the ratio of 52:47:1, at a flow rate of 0.8 ml/min and fluorescence detection (emission-365 nm, excitation-455 nm). Purification efficiency of the aptamers was assessed by plotting measured relative peak area (μV) against various toxin concentrations employing linear regression equation (*y* = *mx*+*c*).

### Statistical Analysis

All experiments were repeated three times with similar conditions to ensure the stability and reliability of the data. Results were presented as the mean value ± standard deviation (SD). Statistical differences between treatments were analyzed by univariate (ANOVA) and Tukey’s test assuming *p* value (*P* < 0.05), (*P* < 0.01), and (*P* < 0.001) followed by Dunnett’s test using Graph Pad Prism6.

## Results

### Optimization of PCR Conditions

In the present study, amplification of aptamer pool using asymmetric PCR was performed. PCR parameters such as number of cycles, annealing temperature, primer concentration were optimized to evade mis-amplification of DNA (Supporting Figures S1A–C). Asymmetric PCR preferentially increased the target ssDNA and decreased the primer dimer as well as non-specific amplification. Optimized reactions inferred a sharp band of expected base pair size (agarose gel analysis) at a combination of 56°C annealing temperature with primer ratio of 1.6:0.4 (Forward: Reverse) after 30 cycles (Supporting Figure S2).

### Modified Immuno Affinity Column Based SELEX

Immuno affinity column based SELEX procedure was performed to identify aptamers against AFB1 as described previously (Supporting Informations 2 and 3). Ten rounds of SELEX were performed, alternating with three rounds of counter SELEX using closely related and other mycotoxins after round 3rd, 5th, and 7th to isolate aptamers that could favorably bind to target toxin with high specificity. After each round of selection, bound aptamer pool was recovered using methanol and concentrated DNA pool was amplified by standardized PCR conditions (see “Optimization of PCR conditions”) and used in subsequent rounds of selection.

**FIGURE 2:**
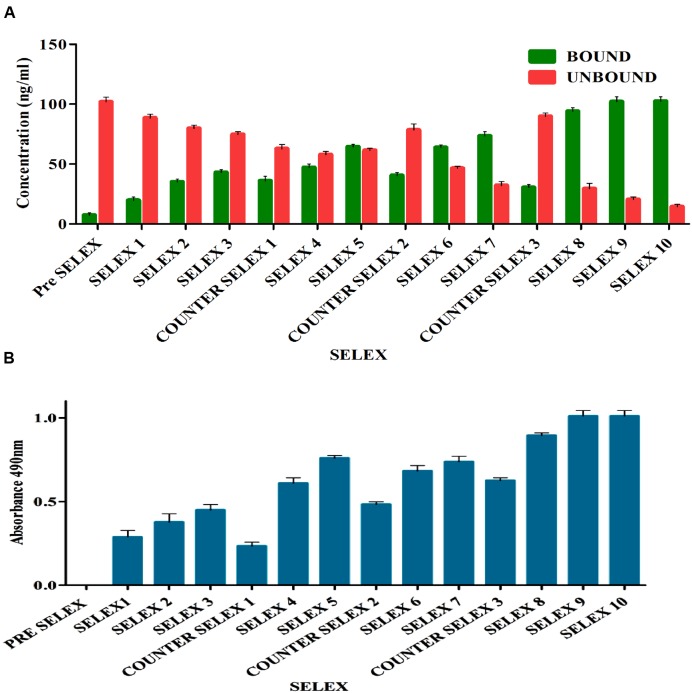
**(A)** Enrichment of bound AFB1-specific aptamers in comparison to unbound ssDNA during immunoaffinity SELEX. The bar graph shows a significant increasing bound aptamer concentration in each election round and nearly steadied in ninth and tenth rounds. Pre SELEX and Counter SELEX steps were incorporated to remove non-specifically ssDNA bound to column components, related and other mycotoxins. **(B)** Observation of AFB1-specific aptamers during immunoaffinity SELEX by enzyme-linked oligonucleotide assay (ELONA). The bar graph illustrates a significant increasing signal over background observed in each selection round and nearly steadied in ninth and tenth rounds. Pre SELEX and Counter SELEX steps were introduced to remove non-specific ssDNA that bound related and other mycotoxins toxins. The experiment was repeated for three times.

Prior to selection, binding saturation of toxin to IA column was analyzed by thin layer chromatography (TLC). Absence of toxin in flow through at concentration of 200 ng indicated optimal binding ability of IA column. During selection, pre SELEX was performed to remove aptamer sequences having affinity to IA column components. PCR amplified unbound flow through was used as aptamer pool in successive rounds of SELEX. After each round of selection, bound and unbound DNA concentrations (**Figure 2A**) were measured and ELONA (**Figure 2B**) was performed to monitor the binding affinity of enriched aptamer pool. As the number of selection rounds progressed, bound DNA concentration and absorbance values gradually increased and remained in steady state during 9th and 10th round of SELEX indicating binding saturation of enriched high affinity aptamer pool. Incorporation of counter SELEX aided in the enrichment of AFB1 specific aptamer pool and elimination of cross reacting aptamers (**Figure 2B**). The stringency of the selection process was controlled by adjusting incubation time and washes. After final round of selection, the purified PCR products were cloned into *E. coli* TOP10 cells.

### Cloning and Sequencing and Structural Analysis of the Selected Aptamers

**FIGURE 3:**
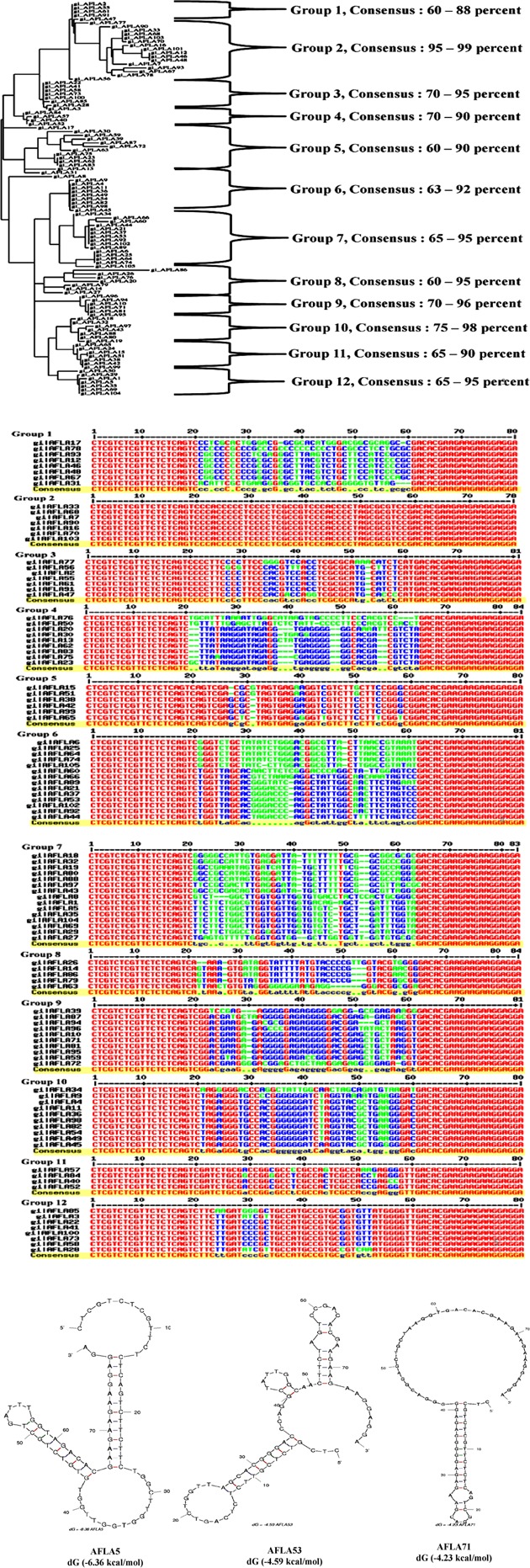
**(A)** Phylogenetic analysis of selected aptamer sequences using Clustal Omega. Each cluster consisting of aptamers sharing common nucleotide sequences and their sequence homology observed was 60–99%. **(B)** Multiple sequence alignment of the obtained aptamers during SELEX procedure. Constant primer regions represented by red color, blue indication moderated similarity and green show the neutral regions. **(C)** Prediction of possible secondary structure of selected aptamer sequences and their free energy (dG) calculated by Mfold software. The resultant secondary structures with the lowest free energy folding are shown here.

A total of 105 positive transformants were sequenced and their structural homology was analyzed using Multalin web based tool. Observations of resultant phylogenetic tree (**Figure 3A**) revealed 12 groups of enriched sequences with 60–99% homology and each group contained certain common sequences: group 1 (3/9 sequences), group 2 (3/7 sequences), group 3 (4/7 sequences), group 4 (2/9 sequences), group 5 (3/9 sequences), group 6 (9/14 sequences), group 7 (7/10 sequences), group 8 (5/8 sequences), group 9 (4/10 sequences), group 10 (7/10 sequences), and group 12 (5/8 sequences), respectively. Jalview analysis (**Figure 3B**) indicated that each group having few base pair length conserved regions which might play an important role in target binding. Possible Secondary structures analyzed using M-fold software showed maximum number of aptamers having typical stem loop structures (Supporting Figure S3). Three aptamers (AFLA5, AFLA53, and AFLA71) with most stable predicted secondary confirmations (lowest Gibbs free energy) are shown in **Figure 3C**. Total 16 aptamers from 12 groups were chosen for further characterization with one to three sequences from each group (Supporting Table 2). Putative QGRS obtained from QGRS Mapper for AFLA5 and AFLA71 showed higher G-scores indicating their G-quadruplex forming ability (**Table 1**; Supporting Table 3).

**Table 1:** Guanine bases that participate in the formation of the G-quadruplex structure are shown in underlined and bold face.

Aptamer number	Sequence (5′ to 3′)
AFLA5	ctcgtctcgttctctcagtcTTCTTCT**GGCTTGGTGGTTGG** TGTGTCTGCTGATTTGGTAgacacgaagaagaaggagga
AFLA71	ctcgtctcgttctctcagtcGGACGAAGAGAGGG**GGAGAGG** **GGGACGG**AGCTGCTAAGGTgacacgaagaagaaggagga

*The small letters in the sequence of Afla5 and Afla71 shows primer binding regions*.

### Aptamer Binding Analysis

**FIGURE 4:**
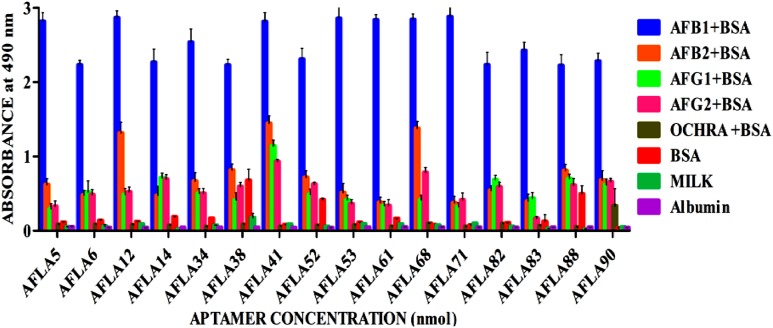
**Determination of specificity of Aflatoxin B1 aptamers using ELONA.** The bar graph show little or no cross-reactivity observed with other mycotoxin-BSA conjugates and negative control (BSA, skimmed milk, albumin). We employed the colorimetric detection system (OPD/H_2_O_2_) for streptavidin peroxidase conjugate to a significant (100X) signal over background.

Relative binding affinity and selectivity of selected aptamers was measured by ELONA. Each aptamer was biotin labeled at the 5′ end and incubated at higher concentration (250 ng/μl) with constant concentration (200 ng) of BSA-AFB1. Unbound aptamer was removed by washing and reaction was developed after incubation with streptavidin conjugated horseradish peroxidase. The same analysis was performed with each of the closely associated and other mycotoxins used in the negative selection to check the specificity of obtained aptamers. As results shown in **Figure 4**, each sequence bound significantly better to AFB1 than to the control species. The DNA sequence library pool showed no significant binding toward the targeted mycotoxin, ruling out possibility of binding by non-specific polyanionic effects.

However, aptamers (AFLA12 – 46% to AFB2, AFLA41 – 54% to AFB2, 42% to AFG1, 33% to AFG2 and AFLA68 – 48% to AFB2, 40% to AFG1 and 30% to AFG2) exhibited affinity toward other aflatoxins when absorbance values of target toxin and aptamer complex was considered as 100 percent. This inference facilitate the possibility of utilizing mentioned aptamers for simultaneous detection two or more aflatoxins.

### Binding Kinetics and Sensitivity Analysis of Selected Aptamers

**FIGURE 5:**
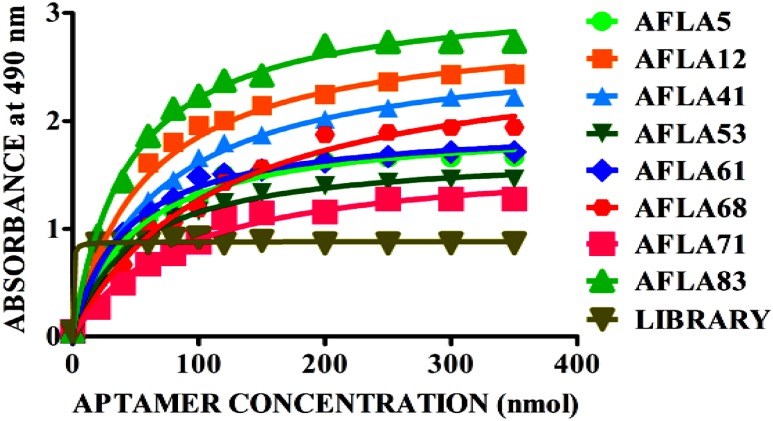
**Binding kinetics of selected aptamer sequences.** Dissociation constants (*K*_d_) of selected aptamer sequences with increasing amounts biotin-labeled ssDNA aptamer (0–350 nM) and a constant amount of AFB1-BSA conjugate (300 ng) for each assay. On the basis of the colorimetric detection system (OPD/H_2_O_2_) for streptavidin peroxidase conjugate to a significant (100X) signal, dissociation constants (*K*_d_) were calculated by non-linear regression analysis.

**FIGURE 6:**
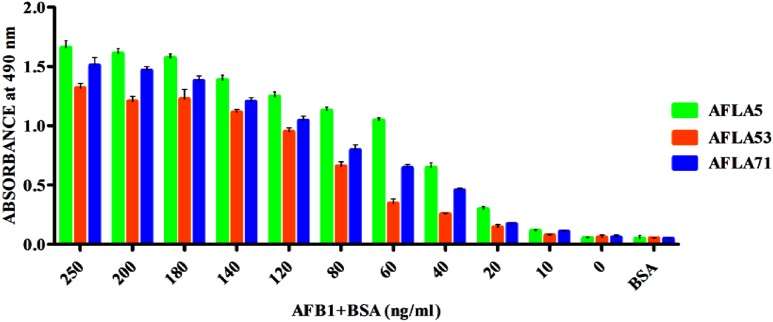
**Determination of Aflatoxin B1 by selected aptamers using ELONA.** The bar graph shows the binding of aptamers with AflatoxinB1-BSA conjugate. We employed the colorimetric detection system (OPD/H_2_O_2_) for streptavidin peroxidase conjugate to a significant (100X) signal over background.

Apparent dissociation constants (*K*_d_) of selected aptamers with high binding affinity and specificity were measured by ELONA. In this assay constant amount of BSA conjugated AFB1 (200 ng) was titrated with various concentrations (0–350 nM) of biotinylated aptamers (**Figure 5**). Tested eight aptamers showed affinities (*K*_d_ = 40–120 nM) (**Table 2**). Three highest affinity binders were chosen for detection of AFB1. As shown in **Figure 6**, aptamer candidates (AFLA5, AFLA53, and AFLA71) bound significantly to increasing concentrations of AFB1-BSA (10–250 ng/ml) and the detection limits (LOD) were approximately (20, 40, and 40 ng/ml), respectively (Supporting Figure S5). Aptamer AFLA5 and AFLA71 were further investigated for affinity purification of AFB1 by spiking studies.

**Table 2:** Selected aptamer sequences and their free energy (dG) and dissociation constants (*K*_d_).

Aptamer number	Sequence (5′ to 3′)	dG value (kcal/mol)	*K*_d_ value (nM)
AFLA5	CTCGTCTCGTTCTCTCAGTCTTCTTCTGGCTTGGTGGTTGG TGTGTCTGCTGATTTGGTAGACACGAAGAAGAAGGAGGA	-6.36	50.45 ± 11.06
AFLA12	CTCGTCTCGTTCTCTCAGTCCGCCCCCCCCGCGCGCGCTT ACGTCTGCTTCCATCCCCGCGACACGAAGAAGAAGGAGGA	-3.93	57.39 ± 18.18
AFLA41	CTCGTCTCGTTCTCTCAGTCTTCTTGATCCCGCTGCCATGC CGTGCGGTGTTATGGGGTTGACACGAAGAAGAAGGAGGA	-3.39	68.24 ± 7.91
AFLA53	CTCGTCTCGTTCTCTCAGTCTGGTTAGCACGGGACCCAGG CTATTGGCAACTTCTAGTCCGACACGAAGAAGAAGGAGGA	-4.59	48.29 ± 9.45
AFLA61	CTCGTCTCGTTCTCTCAGTCCCCTTCCCCTTCCCACGTCCA CCTCGCGCATGCATCTCATGACACGAAGAAGAAGGAGGA	-3.77	41.69 ± 7.39
AFLA68	CTCGTCTCGTTCTCTCAGTCTTCTTCTGGCTTGGTGTTGG TGTGTCTGCTGATTTGGTAGACACGAAGAAGAAGGAGGA	-4.97	113.09 ± 32.22
AFLA71	CTCGTCTCGTTCTCTCAGTCGGACGAAGAGAGGGGGAGAGG GGGACGGAGCTGCTAAGGTGACACGAAGAAGAAGGAGGA	-4.23	85.02 ± 25.74
AFLA83	CTCGTCTCGTTCTCTCAGTCTTATAAGGATAGAGGTGAG GGGGGCACGACGTCTAGACACGAAGAAGAAGGAGGA	-3.80	43.96 ± 6.41

### Purification of Aflatoxin B1 in Food Samples

To demonstrate the potential use of high affinity aptamers, spiking study was conducted using corn sample. Extracted toxin from spiked sample was purified applying biotinylated aptamers. The toxin was then confirmed and quantified by HPLC. A standard curve was obtained by plotting measured relative peak area (μV) against various toxin concentrations (10–250 ng/ml). A linear correlation (*R*^2^ = 0.9963, 0.9942) was obtained for AFLA5 and AFLA71, respectively (**Figures 8A,B**). Considering relative peak area (μV) of standard toxin as 100, estimates for percentage of recovery of toxin from spiked samples were drawn. Aptamer, AFLA5 (LOQ – 53.74 ng) showed recovery of 82.2–96.21% and 78.3–94.22% that of AFLA71 (LOQ – 66.75 ng) with a retention time of 8.88 min (**Figures 9A,B**; **Table 3**). This demonstrates that, selected aptamers from present study can be successfully employed for the detection and purification of Aflatoxin B1 from food and agro commodities.

**FIGURE 7:**
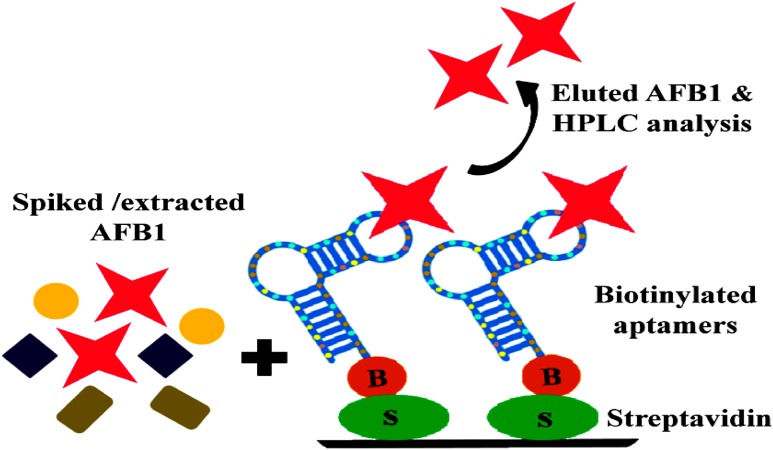
**Schematic illustration of binding and purification of AFB1: Streptavidin bound anti AFB1 biotinylated aptamers incubated with AFB1 solution extracted from spiked corn samples, subsequent elution and HPLC analysis of eluted toxin**.

**FIGURE 8:**
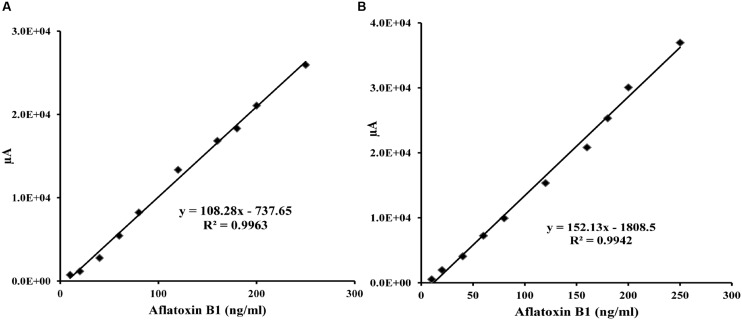
**Calibration curve of the increasing peak area (μA) versus Aflatoxin B1 concentration (0–250 ng/ml) employing streptavidin bound AFLA5 (A)** and AFLA71 **(B)** as recognition element and measured by HPLC method. Data was collected for at least three times.

**FIGURE 9:**
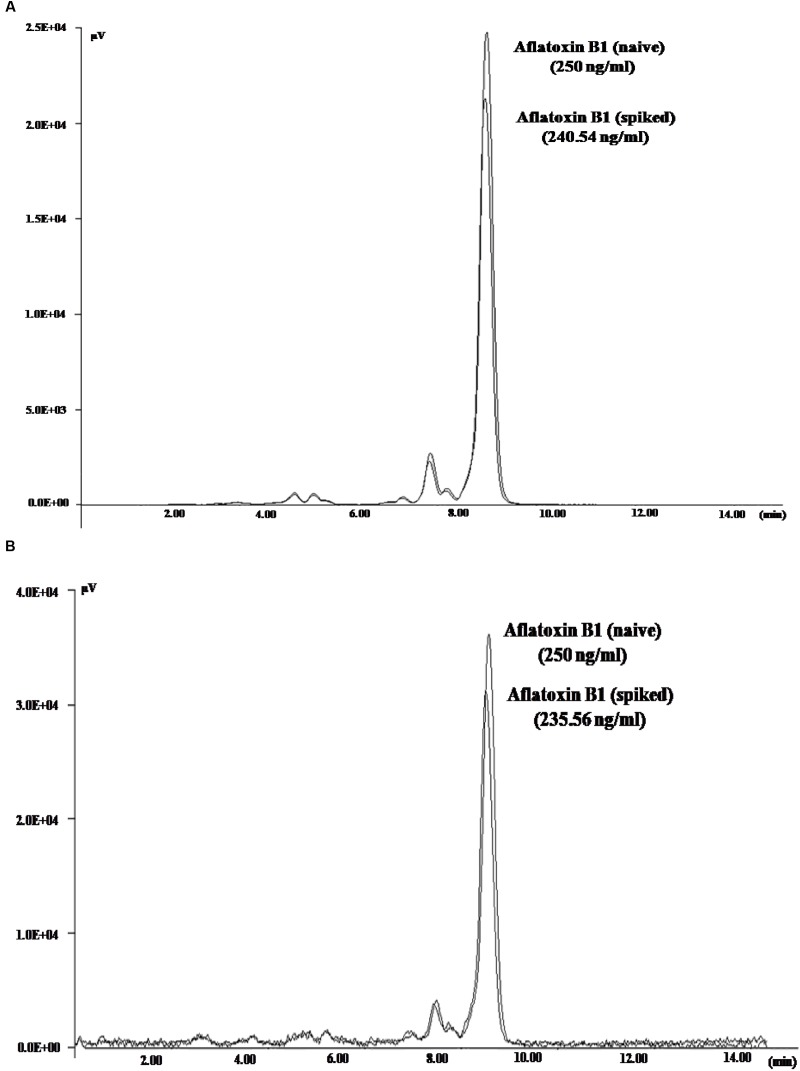
**High Performance Liquid Chromatography Chromatogram depicting the peaks of naive (standard) and eluted AFB1 obtained during spiking and purification studies employing AFLA5 (A)**, AFLA71 **(B)** aptamers. The aptamers was biotinylated and bound to streptavidin coated plate.

**Table 3:** Recovery results of added AFB1 from spiked corn samples obtained by the selected aptamers.

Sample no.	Spiked concentration (ng/ml)	Detected concentration (ng/ml)		Recovery ratio (%)	
		AFLA5	AFLA71	AFLA5	AFLA71
1	10	8.22	7.83	82.2	78.3
2	20	14.32	16.01	71.5	80.05
3	40	32.45	35.34	81.13	88.35
4	60	58.74	55.22	97.9	92.03
5	80	70.56	78.25	88.2	97.81
6	120	111.74	116.84	93.12	97.37
7	140	141.94	135.43	101.38	96.73
8	180	176.43	172.53	98.01	95.85
9	200	200.43	183.45	100.2	91.72
10	250	240.54	235.56	96.21	94.22

*Data was collected for at least three times.*

## Discussion

New generation macromolecules namely aptamers with unique three dimensional structures, high degree of binding affinity and specificity have emerged into an attractive choice in detection of wide range of targets. Aptamers can be exploited through multiple rounds of selection and sequence enrichment, with labeling for visual detection or tethered to solid supports for target capture and detection.

Mycotoxins are small molecules and do not possess any charge or active groups (carboxyl or hydroxyl). Therefore, immobilization of targets and their analogs onto the matrices by complex chemical reactions might alter the native confirmations and possibly lead to reduction in aptamer affinity (Le et al., 2010; McKeague et al., 2010, 2014; Chen et al., 2013, 2014; Ma et al., 2014, 2015; Malhotra et al., 2014). In present report, we describe a modified immunoaffinity based SELEX procedure for selection of aptamers against AFB1. Utilization of antibodies as anchoring molecules in IA column, facilitated us to skip the intricate procedures for activation of AFB1 thus improving the selection efficiency. Further, this approach presents certain unique aspects that are discussed below.

Majority of reported SELEX protocols involve PCR amplification after each round followed by separation of ssDNA by streptavidin coated magnetic beads and alkaline denaturation. During these processes, there are possibilities of losing some real good sequences with high specificity (Paul et al., 2009; Wang et al., 2014). To curtail the mentioned limitation, our strategy employed asymmetric PCR for generation of ssDNA.

In earlier reports, no proper method in monitoring the enrichment of target specific aptamers during SELEX protocol is available. Neglecting this crucial aspect might result in either obtaining cross-reacting aptamers due to less number of rounds or a prolonged SELEX process by unknowingly increasing the number of selection rounds. The SELEX method described in the present study employs ELONA and quantification of bound/unbound aptamer pool after each round of SELEX thereby overcoming this limitation. An added advantage of present SELEX method is incorporation of counter SELEX, which was performed exclusively with other mycotoxins. This approach aided in enrichment of AFB1 specific aptamers by eliminating non-specific sequences.

In another aspect, the highly enriched aptamer pool after 10th round of SELEX was cloned and sequenced to identify 105 aptamer sequences. Phylogenetic and Jalview analysis of the identified aptamer sequences revealed 12 groups of enriched sequences that shared 60–99% homology with few nucleotide conserved regions, hence proving the selection efficiency. Furthermore, secondary structure analysis of each group of aptamers with/without primer binding region revealed minimum change in typical stem-loop structures and low Gibbs free energy (dG) that signify highest binding conformations.

The binding affinity and specificity of representative aptamers with lower dG value were investigated. Eight aptamers showed highest binding affinity toward aflatoxins with little to no reactivity toward other mycotoxins, this substantiated sequence selectivity. Additionally, results also suggested that AFLA12, AFLA41, and AFLA68 having affinity toward other aflatoxins can be used in development of comprehensive assays for simultaneous detection of aflatoxins which coexist in food infested with toxigenic *Aspergillus*. The binding kinetics of highest affinity aptamers was determined. The dissociation constants of AFLA5, AFLA53, and AFLA71 (50.45 ± 11.06, 48.29 ± 9.45, and 85.02 ± 25.74 nM, respectively) were either comparable or better than previously reported aptamers. Therefore, the applicability of lower dissociation constant aptamers in detection of AFB1 was tested by ELONA. Obtained results indicated, AFLA5 (LOD 20 ng/ml) and AFLA71 (LOD 40 ng/ml) can be used as suitable recognition elements in development of AFB1 detection platforms.

Another important facet of the present study was to validate the feasibility of selected aptamers in *in vitro* assays for detection and purification of AFB1 from natural food sample. Spiking studies proved that both aptamers (AFLA5 and AFLA71) can bind and purify toxin with recoveries ranging from 82.2 to 96.21% (LOQ – 53.74 ng) and 78.3 to 94.22% (LOQ – 66.75 ng), respectively. With these finding, we confidently state that aptamers selected by employing the proposed SELEX method show better applicability in detection, purification, and quantification of AFB1 with stability and re-usability.

Key merit of the proposed method state that aptamers generated from presently described protocol can be used for development of reliable, simple, cost effective, and sensitive detection systems for mycotoxins from food and environmental samples during biological emergencies.

## Conclusion

In this study, we present proof-of-concept for successful selection of aptamers exhibiting good specificity toward AFB1 employing immunoaffinity principle via IA columns. On the basis of results in present study, we anticipate that the aptamers AFLA5, AFLA53, and AFLA71 may be used in AFB1 detection in both pure and food samples. Spiking studies in corn samples showed credible recovery which exhibited practical value. Additionally, the description of immuno SELEX enables one to notice the feasibility and flexibility of the procedure in terms of application and obtaining aptamers for rapid detection and purification of other mycotoxins with necessary standardizations in the protocol. The aptamers obtained in the present study finds application in development of rapid, field deployable, cost effective, and highly sensitive platforms for aflatoxin detection.

## Author Contributions

Conceived and designed the experiments: KS, SR, and BM. Performed the experiments: KS. Analyzed the data: KS, SR, and JK. Contributed reagents/materials/analysis tools: JK and SR. Wrote the paper: SR, KS, and BM.

## Conflict of Interest Statement

The authors declare that the research was conducted in the absence of any commercial or financial relationships that could be construed as a potential conflict of interest.

## Footnotes

1 http://multalin.toulouse.inra.fr/multalin/

2 http://unafold.rna.albany.edu/?q=mfold/dna-folding-form

3 http://bioinformatics.ramapo.edu/QGRS/analyze.php
