# Supplementary material for: Immuno Affinity SELEX for Simple, Rapid, and Cost-Effective Aptamer Enrichment and Identification against Aflatoxin B1
Source: Front Microbiol. 2016 Dec 1;7:1909. doi: 10.3389/fmicb.2016.01909 (PMC5130984; doi:10.3389/fmicb.2016.01909)

**Supporting information**

**Title:**

**Immuno affinity SELEX for Simple, Rapid and Cost-Effective Aptamer Enrichment and Identification against Aflatoxin B1.**

**Author’s information:**

Keerthana Setlem **(First author)**

Senior Research Fellow (DRDO-SRF)

Defence Food Research Laboratory (DFRL),

Mysore-570011

Email: [keerthana.set@gmail.com](mailto:keerthana.set@gmail.com)

Bhairab Mondal

Senior Research Fellow, (Inspire fellow, DST)

Defence Food Research Laboratory (DFRL),

Mysore-570011

Email: [bhairabmicro21@gmail.com](mailto:bhairabmicro21@gmail.com)

Dr. Shylaja R **(Corresponding author)**

Scientist ‘D’

Defence Food Research Laboratory (DFRL),

Mysore-570011

Email: [shylajaramlal@gmail.com](mailto:shylajaramlal@gmail.com)

Dr. Joseph Kingston,

Scientist ‘E’

Head, Microbiology division,

Defence Food Research Laboratory (DFRL),

Mysore-570011

Email: josephkingston@yahoo.com

**Supporting information.1:**

**Materials and reagents:**

The single stranded DNA (ssDNA) library and primers, mycotoxins (Aflatoxin B1 (AFB1), Aflatoxin B2 (AFB2), Aflatoxin G1 (AFG1), Aflatoxin G2 (AFG2) and Ochratoxin A (OTA), BSA conjugates (AFB1-BSA, OTA-BSA), potassium phosphate dibasic (K_2_HPO_4_), trizma base (NH_2_C(CH_2_OH)_3_), sodium hydroxide (NaOH), ethylenediaminetetraacetic acid (EDTA), Dulbecco’s phosphate buffered saline (DPBS), bovine serum albumin (BSA), albumin, skimmed milk powder and PCR reagents required for present study were procured from Sigma-Aldrich (India). The other chemicals and organic solvents used in the study were obtained from respective manufactures: Luria-Bertani (LB) broth and LB agar from Himedia (India), PCR purification kit, plasmid extraction kit, and gel extraction kit from Qiagen MiniElute, India, TOPO TA clone PCR Cloning Kit, Streptavidin Conjugate from Invitrogen/Life Technologies, India and *o*-phenylenediamine dihydrochloride (OPD), Hydrogen peroxide (H_2_O_2_) from Bangalore Genie, India. Organic solvents used were of HPLC grade and solutions were prepared with ultrahigh-purity water (Milli-Q). Immunoaffinity columns (IA columns) were procured from LC tech, Germany. Standards of mycotoxins were prepared according to manufactures protocol and diluted from stock solutions to working concentration (1 mg/mL) in absolute methanol (HPLC grade).

**Supporting information.2:**

**Anchoring of AFB1 to immuno affinity column**

Immobilization of AFB1 to IA column was optimized. Initially, the column was pre conditioned with 1X DPBS followed by monitoring the binding efficiency of AFB1 to column at concentrations ranging from 100 ng to 500 ng. Thin Layer Chromatography (TLC) was performed on all the flow through and elutes to examine each variation.

**Supporting information.3:**

**Modified immunoaffinity column based SELEX**

Aptamers exhibiting affinity to AFB1 were selected by iterative rounds of SELEX that consisted of pre SELEX, the denatured aptamer library was passed through an empty IA column to partition aptamers having affinity to column components. This was followed by consecutive rounds of SELEX to screen aptamers specific to IA column bound AFB1. Counter SELEX was introduced after 3^rd^, 5^th^ and 7^th^ round of SELEX to limit aptamers possessing reactivity to closely related and other mycotoxins. Entire process of SELEX rounds was performed under stringent conditions maintaining a constant concentration of AFB1 until the selected aptamer pool reached optimum level.

**Supporting information.3.a:**

**Pre SELEX**

The aptamer library resuspened in 500 µl of 1X BB (5 mM MgCl_2_ in Dulbecco’s phosphate buffered saline, pH 7.4±0.2) was denatured by heating at 94 °C for 10 min and snap cooling in ice for 15 min. The mixture was then loaded onto an empty pre-conditioned IA column and incubated for 30 min at room temperature. The flow through collected from the empty IA column was pooled with one wash and the resultant DNA was used as template library for selection of specific aptamers against Aflatoxin B1 in subsequent rounds of SELEX.

**Supporting information.3.b:**

**Selection of Aflatoxin B1 specific aptamers**

The pre-prepared ssDNA pool (100 ng/µl) from pre SELEX was added onto AFB1 (200 ng/ml) bound IA column and incubated for 1 hr at room temperature in 500 µl of 1X BB. The nucleic acid polymers that remained bound to toxin after two washes were eluted twice with 500 µl of methanol. Each elution was incubated for 5 min prior to collection and pooled together. DNA from pooled elutes was recovered by PCR purification kit following manufacture’s protocol and the concentration of DNA was measured by Nanodrop-2000 (Thermo Scientific, India). The quantified DNA was amplified using earlier standardised PCR conditions, converted to ssDNA and used in subsequent selection rounds. After three successive rounds, counter SELEX was introduced to eliminate non target specific aptamers. Aflatoxins (AFB2, AFG1 and AFG2) were conjugated to BSA molecule according to Cervino *et al*., 2008. Mycotoxin BSA conjugates (200 ng/ml) were immobilized on a nitrocellulose membrane. The membrane was blocked with 3 % bovine serum albumin (BSA) in phosphate-buffered saline (PBS). The denatured ssDNA library (250 ng/μl) was dissolved in 500 μl of 1X BB and incubated with the immobilized mycotoxins BSA conjugates (AFB2, AFG1, AFG2 and OTA) on the membrane at room temperature for 1 hr. After incubation, the nitrocellulose membrane was washed five times with 1 ml of 1X BB. Buffer containing unbound aptamer pool and all wash solutions and were collected in a single vial and recovered by PCR purification kit and dissolved in 50 μl of sterile distilled water and the concentration was measured by Nanodrop-2000 (Thermo Scientific, India). Finally, the DNA was amplified and used as library for further round of SELEX.

**Supporting Information.4:**

**Identification of AFB1 specific aptamers**

Binding affinity of aptamer pool and individual aptamers were evaluated by Enzyme Linked Oligonucleotide Assay (ELONA). For the assay, DNA pool and aptamers were converted to biotin labelled single stranded DNA (Btn-ssDNA) using biotin forward library primer and normal reverse library primer with previously optimised PCR conditions. Mycotoxin BSA conjugates (200 ng/ml) were coated onto the microtiter plate (Nunc) in 100 μl carbonate-bicarbonate buffer (pH 9.6±0.2) and incubated at 37 °C for 60 min in dark followed by three washes with 1X PBST. Blocking of unbound sites was done by 3 % BSA with incubation at 37 °C for 1 hr. Each well was washed thoroughly with 1X PBST prior to the addition of previously prepared Btn–ssDNA pool and incubated for 60 min at 37 °C with intermittent shaking. Later on, the plate was washed thoroughly (3-5 times) and streptavidin-HRP conjugate (1:3000) was added and incubated at room temperature for 45 min followed by through washes (8-10 times) to remove any traces of unbound secondary conjugate. The plate was then treated with developing solution of OPD/H_2_O_2_ for colour development and the reaction was stopped using H_2_SO_4_ (2N). The colour thus developed was quantified by determining the absorbance at 490 nm using Infinite M1000 spectrophotometer (TECAN, India). In order to avoid cross reactivity with BSA molecule, the assay was performed keeping a reaction with BSA as control.

**Supporting information.5:**

**Cloning, sequencing and structural analysis of Aflatoxin specific aptamers**

High affinity aptamer pool from 10^th^ round was cloned using TOPO TA cloning kit following manufacturer’s instructions. The clones thus obtained were confirmed positive by colony PCR using a vector specific primer (M13 forward and reverse primer) (supporting table.1). The positive clones were sequenced by utilizing the in-house sequencing facility (ABI 3500 Genetic Analyzer, Applied Biosystems, USA) at DFRL, India. Obtained sequences were analysed by using online multiple sequence alignment tool, Multalin (<http://multalin.toulouse.inra.fr/multalin/>) (Corpet, 1988). Possible Secondary structures and Gibbs free energy of selected aptamers was predicted using the M-fold software (<http://unafold.rna.albany.edu/?q=mfold/dna-folding-form>) at 26 °C in 150 mM (Na+) and 1 mM (Mg++) (Zuker *et al.,* 2003; SantaLucia *et al.,* 1988). Putative quadruplex forming G Rich sequences (QGRS) of high affinity aptamers using QGRS Mapper software (<http://bioinformatics.ramapo.edu/QGRS/analyze.php>) was predicted (Kikin *et al.,* 2006).

**Supporting Information.6:**

**Determination of dissociation constant (Kd) of selected aptamers:**

High affinity binders were shortlisted for binding kinetic studies. Biotinylation of selected aptamers was done by optimised PCR conditions with biotin forward library and normal reverse library primers. Enzyme Linked Oligonucleotide Assay (ELONA) as described earlier was conducted with Btn-ssDNA. In the assay an increasing concentration (0 to 350 nM) of Btn-ssDNA aptamers were incubated with consistent amount of AFB1-BSA conjugate (200 ng/ml) coated onto the microtiter plate respectively. As described earlier, all steps of the assay were strictly followed. The colour gradation developed was quantified using Infinite M1000 spectrophotometer (TECAN, India) at 490 nm and the obtained values were fitted into the equation y=B_max_*x/(*K_d_*+x) to deduce dissociation constants (*K_d_*) of the aptamers by nonlinear regression analysis using Graph Pad Prism 6 software.

Aptamers with lower *K_d_* values were selected for the development of ELONA to evaluate their detection potential. Various dilutions of AFB1+BSA (250-10 ng/ml) was coated onto microtiter plate and incubated with each biotinylated aptamers of constant concentrations (250 nM) separately. Successive steps of assay were followed according to the procedure mentioned in supporting information.4.

**Supporting Information.7:**

**Determination of Aflatoxin B1 in Spiked food samples**

Corn samples purchased from local market were used in spiking studies. At first, corn was surface sterilized by rinsing with water followed by rapid shaking in 1 % Sodium hypochlorite + 0.1% Tween 20 for 10-15 min and final washing 3-5 times for 5 min in sterile water. Disinfected corn seeds were dried, powdered and stored at 4°C in air tight container to avoid unwanted growth. The aptamers with the best affinity were tested for the application in natural samples. Biotinylated ssDNA was bound onto streptavidin coated microtitre plate and incubated with varying concentrations of AFB1 (10 to 250 ng/ml) for 1 hr at 37 °C. The unbound toxin was removed by washing with 1X BB and bound aflatoxin was eluted with methanol: water (80:20) and analysed by HPLC.

Laterally, varying concentrations AFB1 ranging from 10-250 ng/ml resuspended in methanol was added to 5 gm of powdered corn sample respectively and incubated for 3 hr after through mixing. Toxin extraction from spiked samples was carried with 100 ml of methanol: water (80:20 v/v) by vigorous shaking for 5-8 min and centrifuged at 8000 rpm for 10 min. The supernatants obtained were filtered through Whatman No. 4 filter paper and an equal volume of chloroform was added to the filtrates. This mixture was air dried in dark to remove remnants of organic solvents and resuspended in 1X BB (500 µl) and allowed to react with biotin-ssDNA-streptavidin complex (Btn-ssDNA-Strep) as described previously. The recovery of AFB1 from above mentioned method was confirmed by HPLC analysis, where estimates were drawn from both naive and spiked AFB1 samples. HPLC analysis using JASCO HPLC system (JASCO, UK) containing RP-C18 column (3μm, 250 mm×46 mm) with fluorescence detector at wavelength settings of emission-365 nm and excitation-455nm with a flow rated 0.8ml/min and mobile phase water: acetronitrile: acetic acid (52:47:1) was used to determine AFB1 in the samples (Priyanka et al., 2014).

**Supporting table.1:**

Oligonucleotide sequences of the aptamer library and primers used to amplify the ssDNA library pools, biotin-labeled primer and M13 forward and reverse primers for selection of positive aptamer clones during the selection and characterization of aptamers against Aflatoxin B1. Colour nucleotides represent the primer binding regions flanked on either sides of italicized random region of aptamer library.

| **Name** | **Oligonucleotide sequencec (5'to 3')** |
| --- | --- |
| Aptamer library | **5`**CTCGTCTCGTTCTCTCAGTC*NNNNNNNNNNNNNNNNNNNNNNNNNNNNNNNNNNNNNNNN*GACACGAAGAAGAAGGAGGA3**`** |
| Aptamer forward primer | **5`**CTCGTCTCGTTCTCTCAGTC3**`** |
| Aptamer reverse primer | **5`**TCCTCCTTCTTCTTCGTGTC3**`** |
| Biotin labelled aptamer forward primer | Biotin- **`5**CTCGTCTCGTTCTCTCAGTC3**`** |
| M13 forward primer | **5`**CGCCAGGGTTTTCCCAGTCACGAC3**`** |
| M13 reverse primer | **5`**AGCGGATAACAATTTCACACAGGA3**`** |

**Supporting table.2:**

**Gibbs free energy (dG) of aflatoxin aptamers. Colour nucleotides represent the primer binding regions and bold nucleotides represent the aptamers sequences.**

| **Aptamer ID** | **Oligonucleotide sequence (5' to 3')** | **dG value (kcal/mol)** |
| --- | --- | --- |
| **AFLA6** | **CTCGTCTCGTTCTCTCAGTCGGGTCTGCTATATCTGGGACGGCGTTACTTAACCGTAAATGACACGAAGAAGAAGGAGGA** | **-3.69 kcal/mol** |
| **AFLA12** | **CTCGTCTCGTTCTCTCAGTCCGCCCCCCCCGCGCGCGCTTACGTCTGCTTCCATCCCCGCGACACGAAGAAGAAGGAGGA** | **-3.17 kcal/mol** |
| **AFLA14** | **CTCGTCTCGTTCTCTCAGTCAGTAAAGTGATAGGTATTTTATGTACCCCGGTACGAACGGGACACGAAGAAGAAGGAGGA** | **-3.77 kcal/mol** |
| **AFLA34** | **CTCGTCTCGTTCTCTCAGTCAAGGGGGACCCAGGCTATTGGCAACTAGCAGATGTAAGATGACACGAAGAAGAAGGAGGA** | **-4.10 kcal/mol** |
| **AFLA38** | **CTCGTCTCGTTCTCTCAGTCAGTCGAGCGCTAGTGAGGAGGTCGTCTTCCTTCCGGGCGGACACGAAGAAGAAGGAGGA** | **-3.67 kcal/mol** |
| **AFLA41** | **CTCGTCTCGTTCTCTCAGTCTTCTTGATCCCGCTGCCATGCCGTGCGGTGTTATGGGGTTGACACGAAGAAGAAGGAGGA** | **-3.39 kcal/mol** |
| **AFLA52** | **CTCGTCTCGTTCTCTCAGTCGATCTGCGACCGGCGCCTCGCCACTCGCCACCGAGGGGTTGACACGAAGAAGAAGGAGGA** | **-7.57 kcal/mol** |
| **AFLA61** | **CTCGTCTCGTTCTCTCAGTCCCCTTCCCCTTCCCACGTCCACCTCGCGCATGCATCTCATGACACGAAGAAGAAGGAGGA** | **-2.50 kcal/mol** |
| **AFLA68** | **CTCGTCTCGTTCTCTCAGTCCCACCCCCTCCCCTCGCCGTCCCACCCCTAGCGCGTCCGACACGAAGAAGAAGGAGGA** | **-2.15 kcal/mol** |
| **AFLA82** | **CTCGTCTCGTTCTCTCAGTCTAGAGGGTGCCACGGGGGGATCTAGGTACGCTGAAGGGACGACACGAAGAAGAAGGAGGA** | **-3.07 kcal/mol** |
| **AFLA83** | **CTCGTCTCGTTCTCTCAGTCTTATAAGGATAGAGGTGAGGGGGGCACGACGTCTAGACACGAAGAAGAAGGAGGA** | **-1.59 kcal/mol** |
| **AFLA88** | **CTCGTCTCGTTCTCTCAGTCGGCCGCCATAGTGAGGATTATGCTTTTTGCGGCGCCAGGGGACACGAAGAAGAAGGAGGA** | **-3.73 kcal/mol** |
| **AFLA90** | **CTCGTCTCGTTCTCTCAGTCCCCGCCCCCCCCCCTGCGCCCTCCTGCCCCGTGGCCAGCCGACACGAAGAAGAAGGAGGA** | **-5.39 kcal/mol** |

**Supplemental Table.3**:

Prediction of G-score by QGRS mapper for aptamers AFLA5, AFLA71 with overlaps. Underlined ‘G’ characters are predicted to form G-quadruplex structure. G-score is the meaning of tendency for G-quadruplex structures formations. Search parameters: QGRS Max Length: 30; Min G-Group size: 2; Loop size: 0 to 36; Loop search string: QGRS sequences found (overlaps included).

| **Aptamer number** | **Position** | **Length** | **QGRS** | **G-Score** |
| --- | --- | --- | --- | --- |
| **AFLA5** | **28** | **14** | **GG**CTT**GG**T**GG**TT**GG** | **19** |
|  | **33** | **26** | **GG**T**GG**TT**GG**TGTGTCTGCTGATTT**GG** | **7** |
| **AFLA71** | **21** | **21** | **GG**ACGAAGAGA**GGGG**GAGA**GG** | **12** |
|  | **21** | **21** | **GG**ACGAAGAGA**GG**G**GG**AGA**GG** | **13** |
|  | **21** | **21** | **GG**ACGAAGAGAG**GGGG**AGA**GG** | **11** |
|  | **21** | **22** | **GG**ACGAAGAGA**GGGG**GAGAG**GG** | **12** |
|  | **21** | **22** | **GG**ACGAAGAGA**GG**G**GG**AGAG**GG** | **13** |
|  | **21** | **22** | **GG**ACGAAGAGAG**GGGG**AGAG**GG** | **11** |
|  | **21** | **23** | **GG**ACGAAGAGA**GGGG**GAGAGG**GG** | **12** |
|  | **21** | **23** | **GG**ACGAAGAGA**GG**G**GG**AGAGG**GG** | **13** |
|  | **21** | **23** | **GG**ACGAAGAGA**GG**GGGAGA**GGGG** | **12** |
|  | **21** | **23** | **GG**ACGAAGAGAG**GGGG**AGAGG**GG** | **11** |
|  | **21** | **23** | **GG**ACGAAGAGAG**GG**GGAGA**GGGG** | **11** |
|  | **21** | **23** | **GG**ACGAAGAGAGG**GG**GAGA**GGGG** | **10** |
|  | **21** | **23** | **GG**ACGAAGAGAGGG**GG**AGA**GGGG** | **9** |
|  | **21** | **24** | **GG**ACGAAGAGA**GGGG**GAGAGGG**GG** | **12** |
|  | **21** | **24** | **GG**ACGAAGAGA**GG**G**GG**AGAGGG**GG** | **13** |
|  | **21** | **24** | **GG**ACGAAGAGA**GG**GGGAGA**GG**G**GG** | **13** |
|  | **21** | **24** | **GG**ACGAAGAGA**GG**GGGAGAG**GGGG** | **12** |
|  | **21** | **24** | **GG**ACGAAGAGAG**GGGG**AGAGGG**GG** | **11** |
|  | **21** | **24** | **GG**ACGAAGAGAG**GG**GGAGA**GG**G**GG** | **12** |
|  | **21** | **24** | **GG**ACGAAGAGAG**GG**GGAGAG**GGGG** | **11** |
|  | **21** | **24** | **GG**ACGAAGAGAGG**GG**GAGA**GG**G**GG** | **11** |
|  | **21** | **24** | **GG**ACGAAGAGAGG**GG**GAGAG**GGGG** | **10** |
|  | **21** | **24** | **GG**ACGAAGAGAGGG**GG**AGA**GG**G**GG** | **10** |
|  | **21** | **24** | **GG**ACGAAGAGAGGG**GG**AGAG**GGGG** | **9** |
|  | **21** | **28** | **GG**ACGAAGAGA**GGGG**GAGAGGGGGAC**GG** | **10** |
|  | **21** | **28** | **GG**ACGAAGAGA**GG**G**GG**AGAGGGGGAC**GG** | **12** |
|  | **21** | **28** | **GG**ACGAAGAGA**GG**GGGAGA**GG**GGGAC**GG** | **17** |
|  | **21** | **28** | **GG**ACGAAGAGA**GG**GGGAGAG**GG**GGAC**GG** | **16** |
|  | **21** | **28** | **GG**ACGAAGAGA**GG**GGGAGAGG**GG**GAC**GG** | **15** |
|  | **21** | **28** | **GG**ACGAAGAGA**GG**GGGAGAGGG**GG**AC**GG** | **14** |
|  | **21** | **28** | **GG**ACGAAGAGAG**GGGG**AGAGGGGGAC**GG** | **11** |
|  | **21** | **28** | **GG**ACGAAGAGAG**GG**GGAGA**GG**GGGAC**GG** | **16** |
|  | **21** | **28** | **GG**ACGAAGAGAG**GG**GGAGAG**GG**GGAC**GG** | **15** |
|  | **21** | **28** | **GG**ACGAAGAGAG**GG**GGAGAGG**GG**GAC**GG** | **14** |
|  | **21** | **28** | **GG**ACGAAGAGAG**GG**GGAGAGGG**GG**AC**GG** | **13** |
|  | **21** | **28** | **GG**ACGAAGAGAGG**GG**GAGA**GG**GGGAC**GG** | **14** |
|  | **21** | **28** | **GG**ACGAAGAGAGG**GG**GAGAG**GG**GGAC**GG** | **14** |
|  | **21** | **28** | **GG**ACGAAGAGAGG**GG**GAGAGG**GG**GAC**GG** | **13** |
|  | **21** | **28** | **GG**ACGAAGAGAGG**GG**GAGAGGG**GG**AC**GG** | **12** |
|  | **21** | **28** | **GG**ACGAAGAGAGGG**GG**AGA**GG**GGGAC**GG** | **12** |
|  | **21** | **28** | **GG**ACGAAGAGAGGG**GG**AGAG**GG**GGAC**GG** | **13** |
|  | **21** | **28** | **GG**ACGAAGAGAGGG**GG**AGAGG**GG**GAC**GG** | **12** |
|  | **21** | **28** | **GG**ACGAAGAGAGGG**GG**AGAGGG**GG**AC**GG** | **11** |
|  | **21** | **28** | **GG**ACGAAGAGAGGGGGAGA**GGGG**GAC**GG** | **4** |
|  | **21** | **28** | **GG**ACGAAGAGAGGGGGAGA**GG**G**GG**AC**GG** | **5** |
|  | **21** | **28** | **GG**ACGAAGAGAGGGGGAGAG**GGGG**AC**GG** | **3** |
|  | **32** | **12** | **GG**G**GG**AGA**GGGG** | **18** |
|  | **32** | **13** | **GGGG**GAGA**GG**G**GG** | **17** |
|  | **32** | **13** | **GG**G**GG**AGA**GG**G**GG** | **19** |
|  | **32** | **13** | **GG**G**GG**AGAG**GGGG** | **17** |
|  | **32** | **17** | **GGGG**GAGA**GG**GGGAC**GG** | **16** |
|  | **32** | **17** | **GGGG**GAGAG**GG**GGAC**GG** | **16** |
|  | **32** | **17** | **GGGG**GAGAGG**GG**GAC**GG** | **15** |
|  | **32** | **17** | **GGGG**GAGAGGG**GG**AC**GG** | **14** |
|  | **32** | **17** | **GG**G**GG**AGA**GG**GGGAC**GG** | **17** |
|  | **32** | **17** | **GG**G**GG**AGAG**GG**GGAC**GG** | **18** |
|  | **32** | **17** | **GG**G**GG**AGAGG**GG**GAC**GG** | **17** |
|  | **32** | **17** | **GG**G**GG**AGAGGG**GG**AC**GG** | **16** |
|  | **32** | **17** | **GG**GGGAGA**GGGG**GAC**GG** | **15** |
|  | **32** | **17** | **GG**GGGAGA**GG**G**GG**AC**GG** | **16** |
|  | **32** | **17** | **GG**GGGAGAG**GGGG**AC**GG** | **14** |
|  | **32** | **28** | **GGGG**GAGA**GG**GGGACGGAGCTGCTAA**GG** | **5** |
|  | **32** | **28** | **GGGG**GAGAG**GG**GGACGGAGCTGCTAA**GG** | **6** |
|  | **32** | **28** | **GGGG**GAGAGG**GG**GACGGAGCTGCTAA**GG** | **7** |
|  | **32** | **28** | **GGGG**GAGAGGG**GG**ACGGAGCTGCTAA**GG** | **8** |
|  | **32** | **28** | **GGGG**GAGAGGGGGAC**GG**AGCTGCTAA**GG** | **10** |
|  | **32** | **28** | **GG**G**GG**AGA**GG**GGGACGGAGCTGCTAA**GG** | **6** |
|  | **32** | **28** | **GG**G**GG**AGAG**GG**GGACGGAGCTGCTAA**GG** | **7** |
|  | **32** | **28** | **GG**G**GG**AGAGG**GG**GACGGAGCTGCTAA**GG** | **8** |
|  | **32** | **28** | **GG**G**GG**AGAGGG**GG**ACGGAGCTGCTAA**GG** | **9** |
|  | **32** | **28** | **GG**G**GG**AGAGGGGGAC**GG**AGCTGCTAA**GG** | **12** |
|  | **32** | **28** | **GG**GGGAGA**GGGG**GACGGAGCTGCTAA**GG** | **7** |
|  | **32** | **28** | **GG**GGGAGA**GG**G**GG**ACGGAGCTGCTAA**GG** | **9** |
|  | **32** | **28** | **GG**GGGAGA**GG**GGGAC**GG**AGCTGCTAA**GG** | **17** |
|  | **32** | **28** | **GG**GGGAGAG**GGGG**ACGGAGCTGCTAA**GG** | **8** |
|  | **32** | **28** | **GG**GGGAGAG**GG**GGAC**GG**AGCTGCTAA**GG** | **16** |
|  | **32** | **28** | **GG**GGGAGAGG**GG**GAC**GG**AGCTGCTAA**GG** | **15** |
|  | **32** | **28** | **GG**GGGAGAGGG**GG**AC**GG**AGCTGCTAA**GG** | **14** |
|  | **33** | **12** | **GGGG**AGA**GG**G**GG** | **18** |
|  | **33** | **16** | **GGGG**AGA**GG**GGGAC**GG** | **16** |
|  | **33** | **16** | **GGGG**AGAG**GG**GGAC**GG** | **17** |
|  | **33** | **16** | **GGGG**AGAGG**GG**GAC**GG** | **16** |
|  | **33** | **16** | **GGGG**AGAGGG**GG**AC**GG** | **15** |
|  | **33** | **16** | **GG**GGAGA**GGGG**GAC**GG** | **16** |
|  | **33** | **16** | **GG**GGAGA**GG**G**GG**AC**GG** | **17** |
|  | **33** | **16** | **GG**GGAGAG**GGGG**AC**GG** | **15** |
|  | **33** | **27** | **GGGG**AGA**GG**GGGACGGAGCTGCTAA**GG** | **5** |
|  | **33** | **27** | **GGGG**AGAG**GG**GGACGGAGCTGCTAA**GG** | **6** |
|  | **33** | **27** | **GGGG**AGAGG**GG**GACGGAGCTGCTAA**GG** | **7** |
|  | **33** | **27** | **GGGG**AGAGGG**GG**ACGGAGCTGCTAA**GG** | **8** |
|  | **33** | **27** | **GGGG**AGAGGGGGAC**GG**AGCTGCTAA**GG** | **11** |
|  | **33** | **27** | **GG**GGAGA**GGGG**GACGGAGCTGCTAA**GG** | **7** |
|  | **33** | **27** | **GG**GGAGA**GG**G**GG**ACGGAGCTGCTAA**GG** | **9** |
|  | **33** | **27** | **GG**GGAGA**GG**GGGAC**GG**AGCTGCTAA**GG** | **17** |
|  | **33** | **27** | **GG**GGAGAG**GGGG**ACGGAGCTGCTAA**GG** | **8** |
|  | **33** | **27** | **GG**GGAGAG**GG**GGAC**GG**AGCTGCTAA**GG** | **16** |
|  | **33** | **27** | **GG**GGAGAGG**GG**GAC**GG**AGCTGCTAA**GG** | **15** |
|  | **33** | **27** | **GG**GGAGAGGG**GG**AC**GG**AGCTGCTAA**GG** | **14** |
|  | **34** | **15** | **GG**GAGA**GGGG**GAC**GG** | **17** |
|  | **34** | **15** | **GG**GAGA**GG**G**GG**AC**GG** | **18** |
|  | **34** | **15** | **GG**GAGAG**GGGG**AC**GG** | **16** |
|  | **34** | **26** | **GG**GAGA**GGGG**GACGGAGCTGCTAA**GG** | **7** |
|  | **34** | **26** | **GG**GAGA**GG**G**GG**ACGGAGCTGCTAA**GG** | **9** |
|  | **34** | **26** | **GG**GAGA**GG**GGGAC**GG**AGCTGCTAA**GG** | **16** |
|  | **34** | **26** | **GG**GAGAG**GGGG**ACGGAGCTGCTAA**GG** | **8** |
|  | **34** | **26** | **GG**GAGAG**GG**GGAC**GG**AGCTGCTAA**GG** | **16** |
|  | **34** | **26** | **GG**GAGAGG**GG**GAC**GG**AGCTGCTAA**GG** | **15** |
|  | **34** | **26** | **GG**GAGAGGG**GG**AC**GG**AGCTGCTAA**GG** | **14** |
|  | **35** | **14** | **GG**AGA**GGGG**GAC**GG** | **18** |
|  | **35** | **14** | **GG**AGA**GG**G**GG**AC**GG** | **19** |
|  | **35** | **14** | **GG**AGAG**GGGG**AC**GG** | **17** |
|  | **35** | **25** | **GG**AGA**GGGG**GACGGAGCTGCTAA**GG** | **7** |
|  | **35** | **25** | **GG**AGA**GG**G**GG**ACGGAGCTGCTAA**GG** | **9** |
|  | **35** | **25** | **GG**AGA**GG**GGGAC**GG**AGCTGCTAA**GG** | **15** |
|  | **35** | **25** | **GG**AGAG**GGGG**ACGGAGCTGCTAA**GG** | **8** |
|  | **35** | **25** | **GG**AGAG**GG**GGAC**GG**AGCTGCTAA**GG** | **16** |
|  | **35** | **25** | **GG**AGAGG**GG**GAC**GG**AGCTGCTAA**GG** | **15** |
|  | **35** | **25** | **GG**AGAGGG**GG**AC**GG**AGCTGCTAA**GG** | **14** |
|  | **40** | **20** | **GGGG**GAC**GG**AGCTGCTAA**GG** | **12** |
|  | **40** | **20** | **GG**G**GG**AC**GG**AGCTGCTAA**GG** | **13** |
|  | **41** | **19** | **GGGG**AC**GG**AGCTGCTAA**GG** | **12** |

**Supporting figure.S1.a:**

Optimization of number of cycles.  **L1**: DNA ladder (50 bp), **L2**:20 cycles, **L3**:25 cycles, **L4**: 30 cycles, **L5**: 35 cycles.


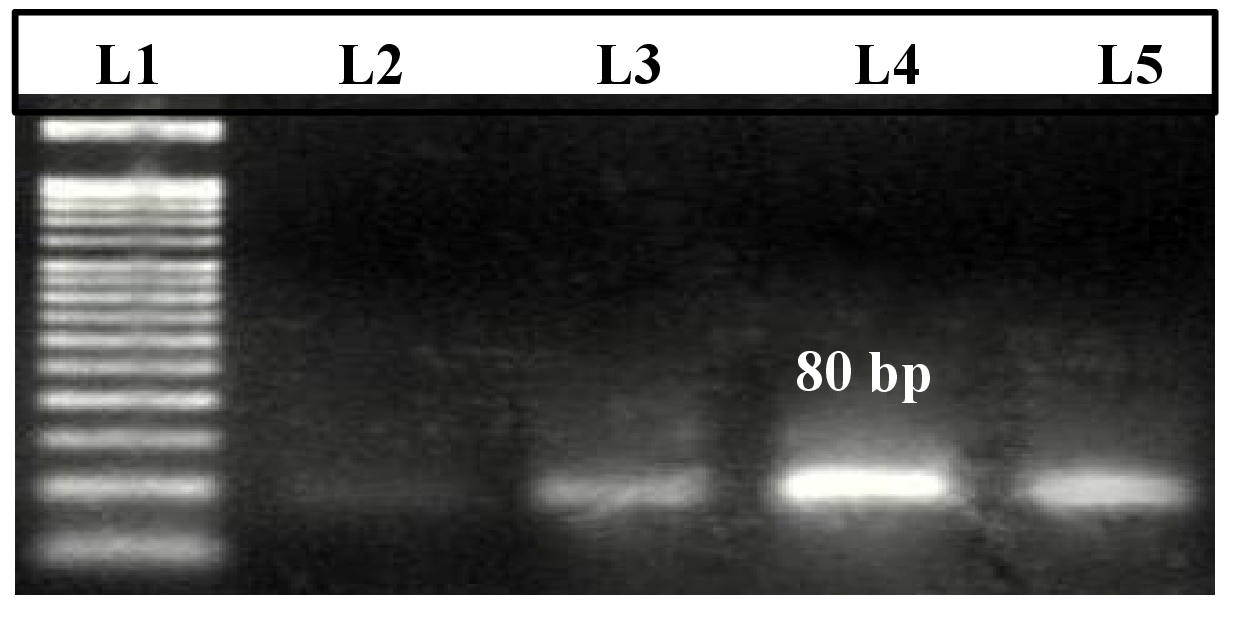


**Supporting figure.S1.b:**

**O**ptimization of annealing temperature. **L1**: 52 °C, **L2**: 54 °C, **L3**: 56 °C, **L4**: 58 °C, **L5**: 60 °C, **L6**: DNA ladder (50 bp).


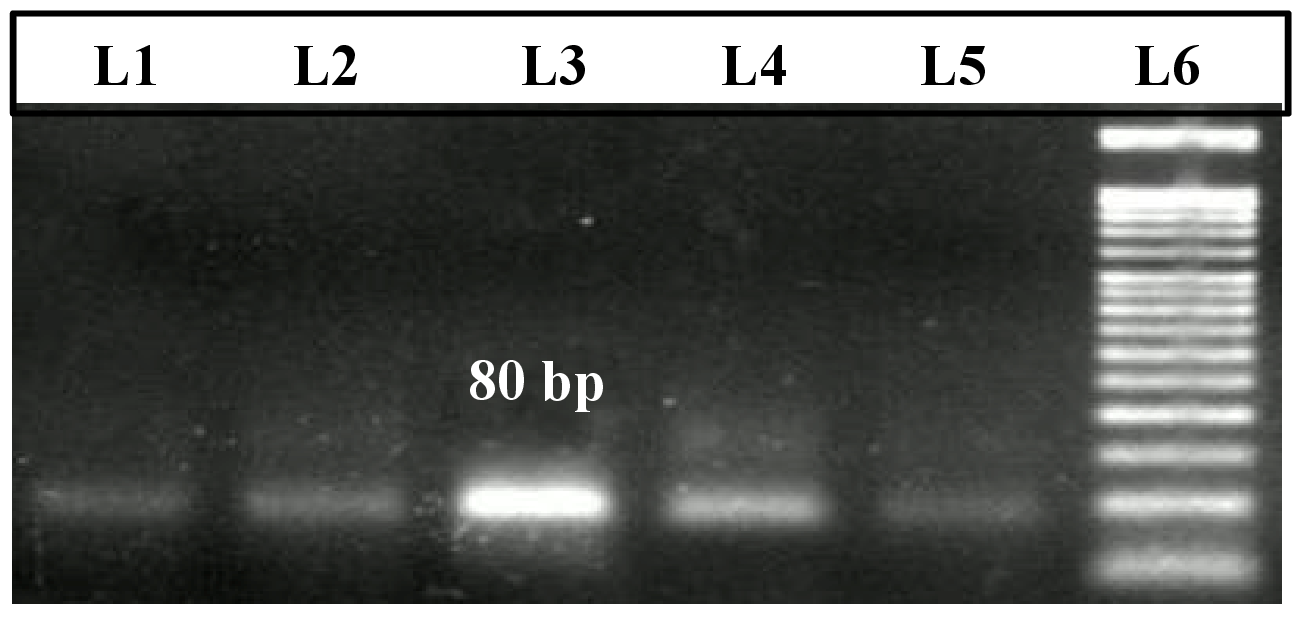


**Supporting figure.S1.c:**

Amplification of aptamer pool with various ratio of forward: reverse primer. **L1**: 1.1:0.9, **L2**: 1.2:0.8, **L3**: 1.3:0.7, **L4**: 1.4:0.6, **L5**: 1.5:0.5, **L6**: 1.6:0.4, **L7**: 1.7:0.3, **L8**: 1.8:0.2, **L9**: 1.9:0.1. **L10**: 2.0:0.0, **L11**: DNA ladder (50 bp).


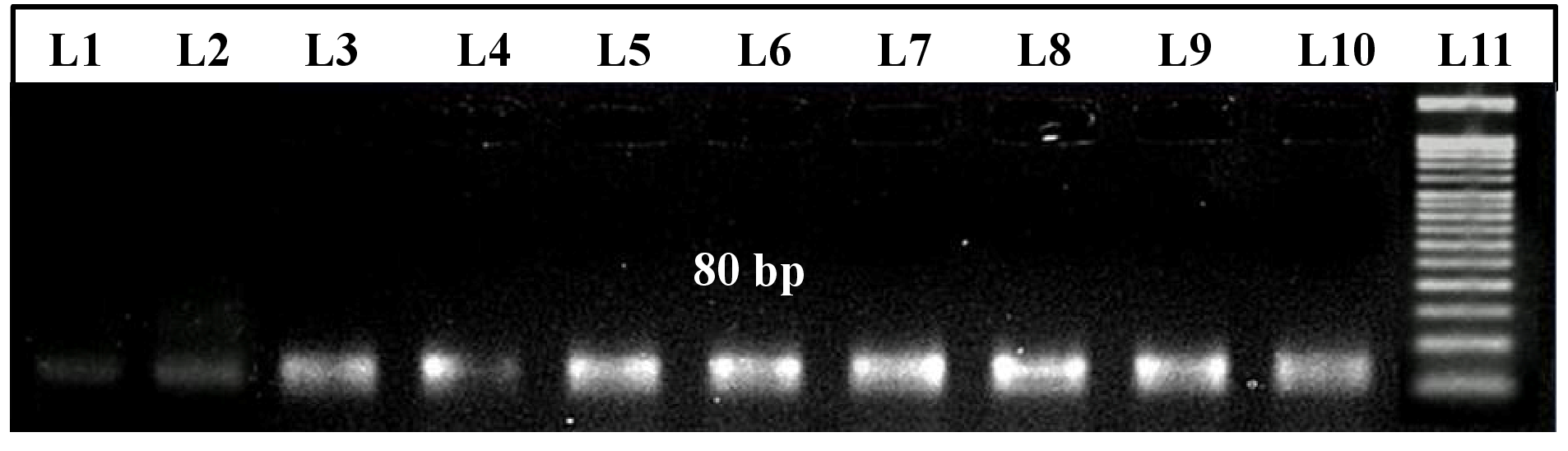


**Supporting figure.S2:**

Amplification of aptamer pool with optimised conditions.

**No. of cycles:** 30, **Annealing temperature:** 56 ℃, **Primer concentration (Fwd:Rev):** 1.6:0.4.


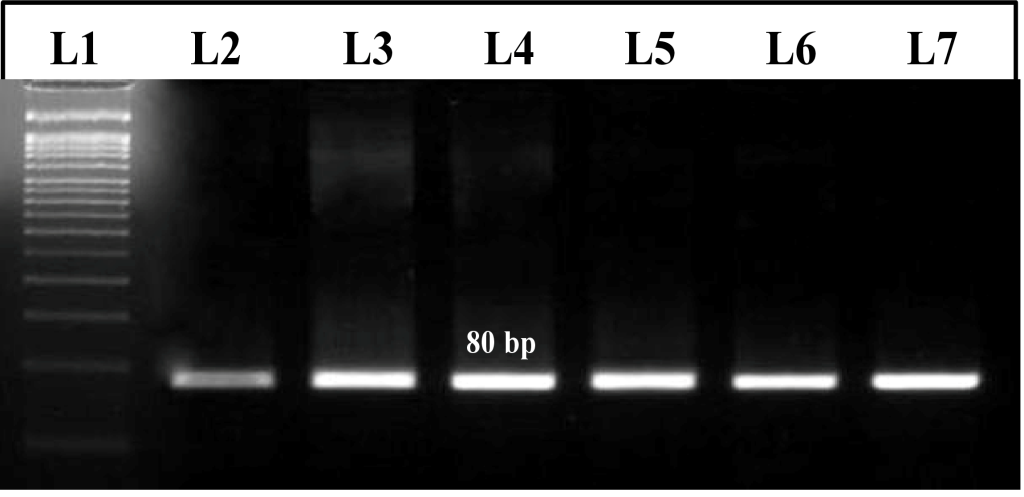


**Supporting figure.S3:**

Prediction of possible secondary structure of selected aptamer sequences and their free energy (dG) calculated by Mfold software. The resultant secondary structures with the lowest free energy folding are shown.

**AFLA6 (-3.69 kcal/mol)**

**AFLA12 (-3.17 kcal/mol)**

**AFLA14 (-3.77 kcal/mol)**

**AFLA34 (-4.10 kcal/mol)**

**AFLA38 (-3.67 kcal/mol)**

**AFLA52 (-7.57 kcal/mol)**

**AFLA61 (-2.50 kcal/mol)**

**AFLA68 (-2.15 kcal/mol)**


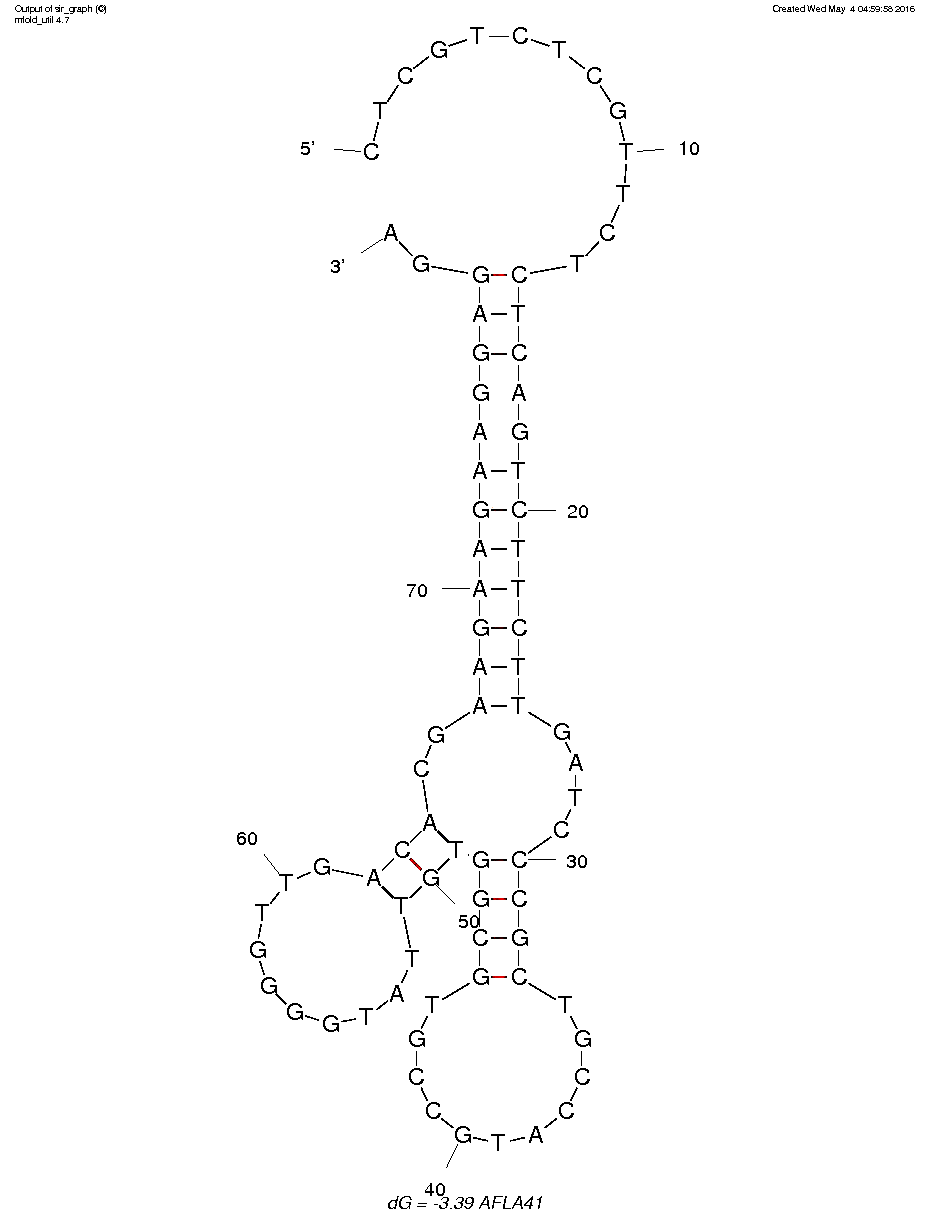


**AFLA41 (-3.39 kcal/mol)**


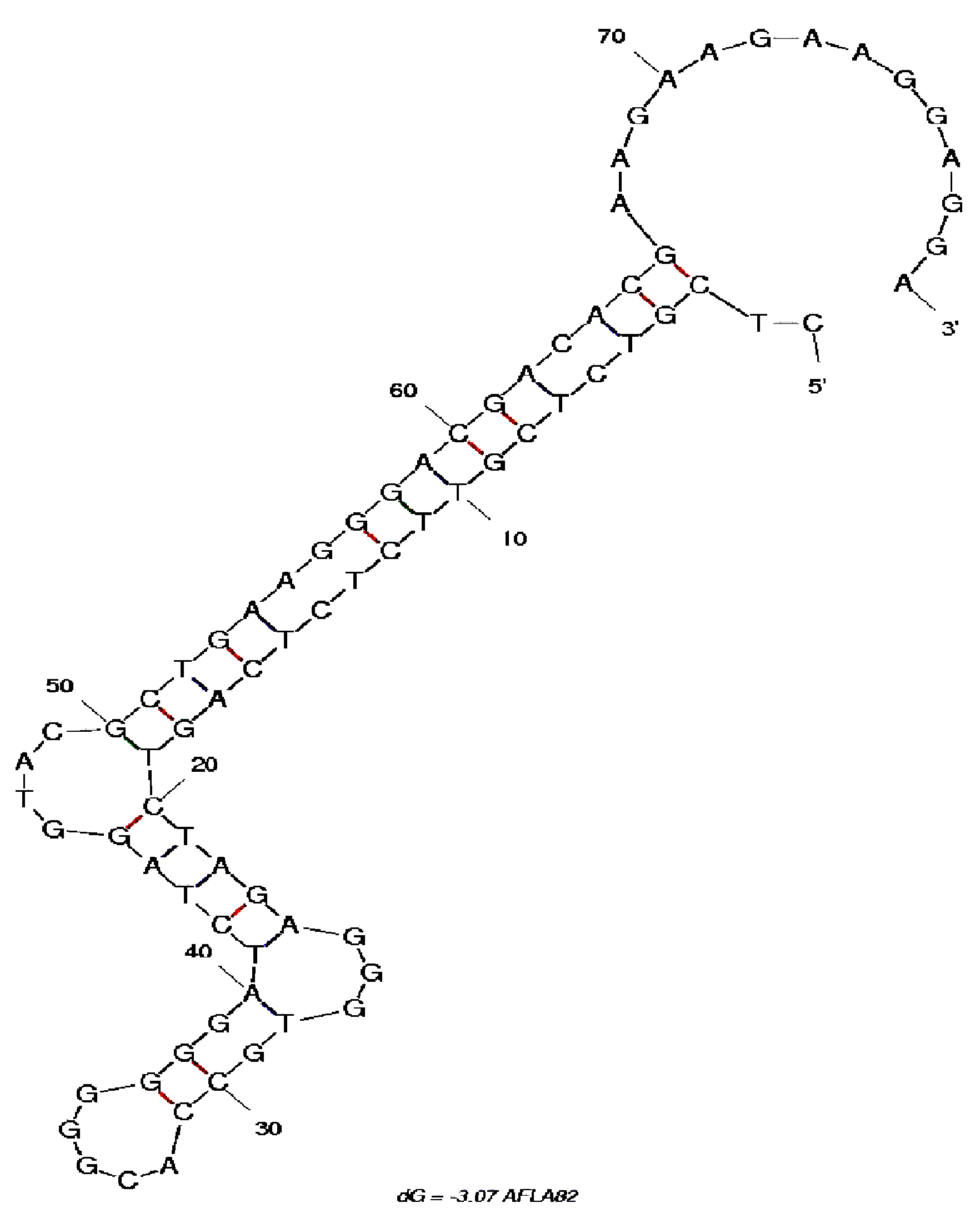


**AFLA82 (-3.07 kcal/mol)**


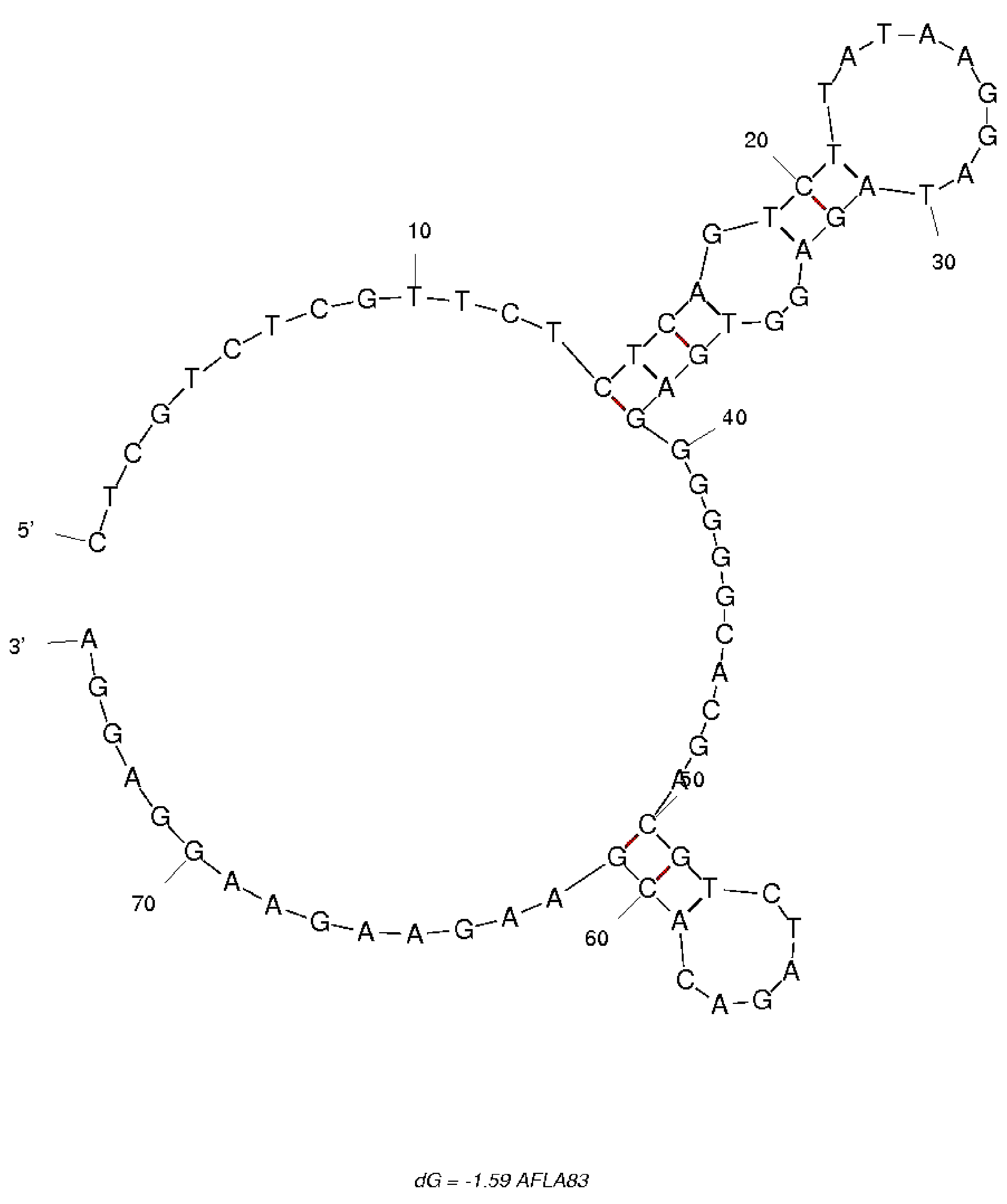


**AFLA83 (-1.59 kcal/mol)**


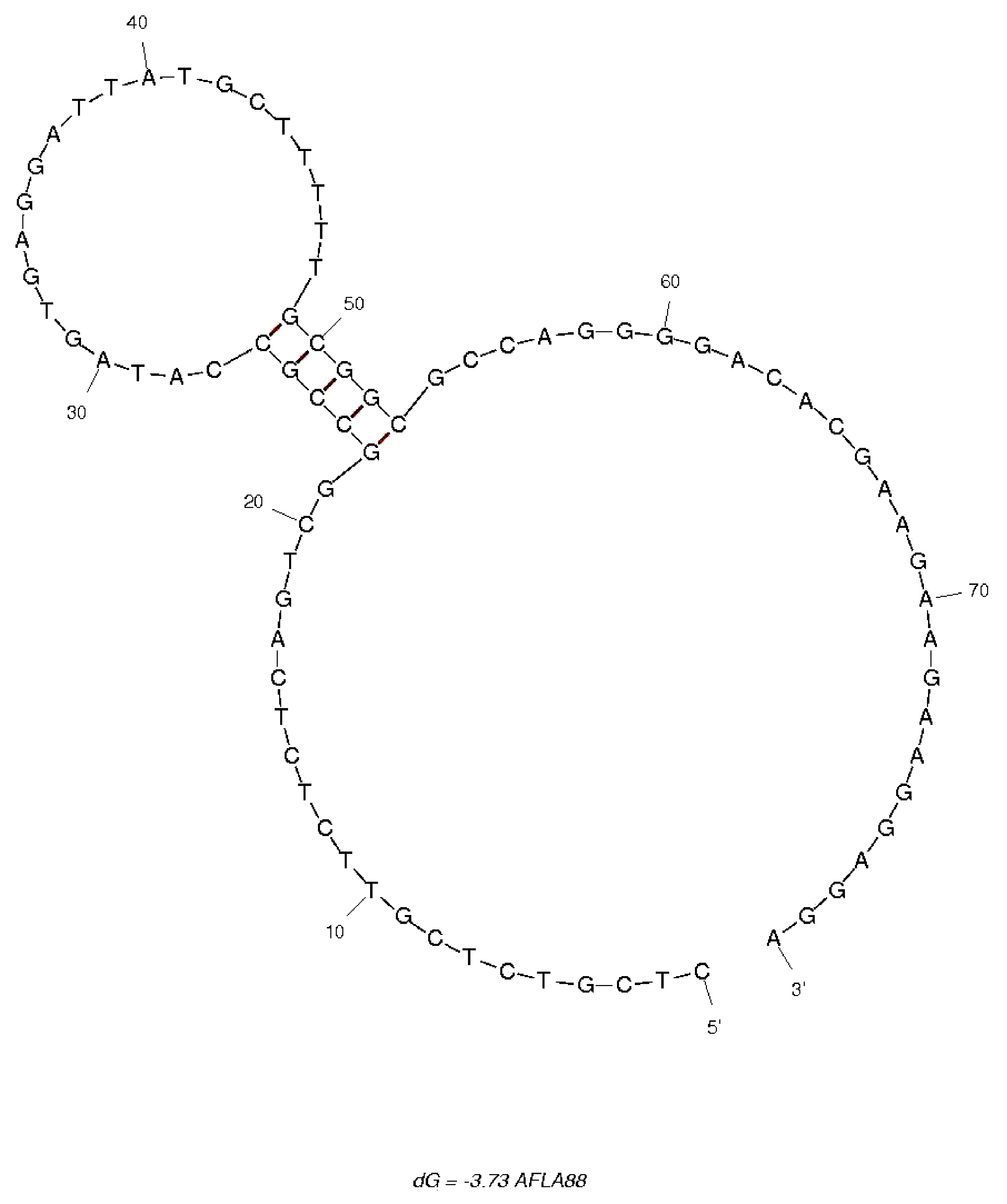


**AFLA88 (-3.75 kcal/mol)**


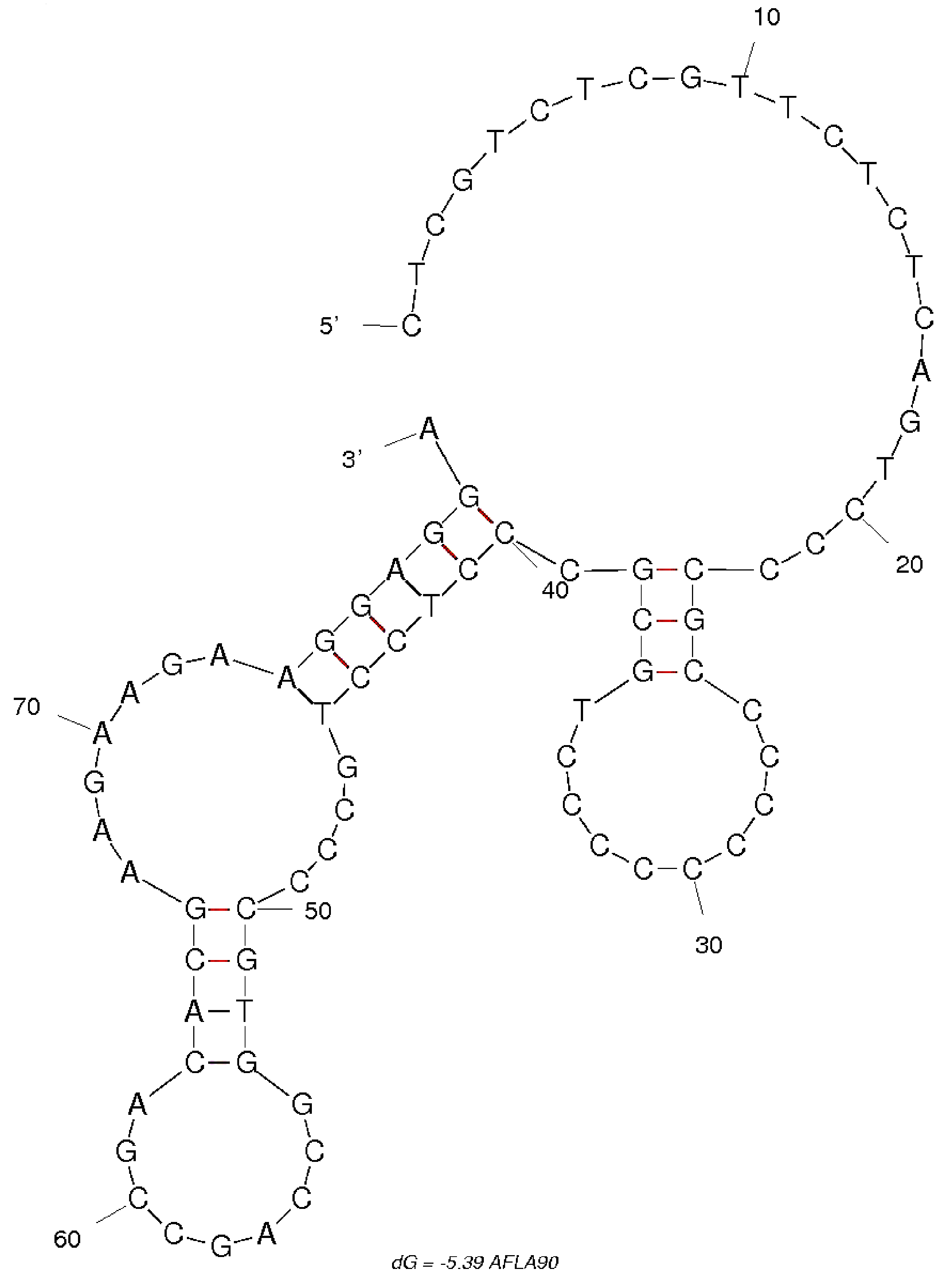


**AFLA90 (-5.39 kcal/mol)**


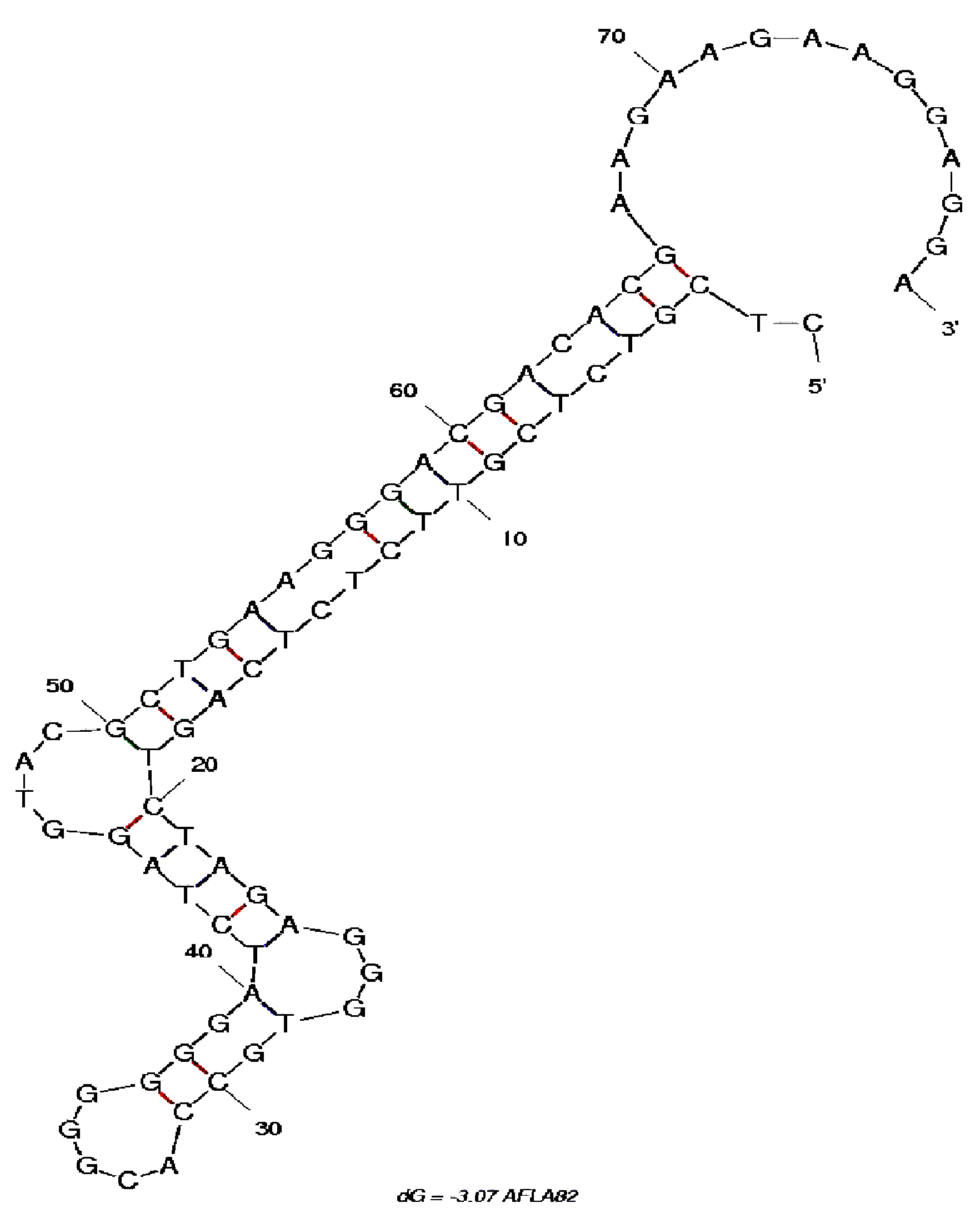


**AFLA82 (-3.07 kcal/mol)**


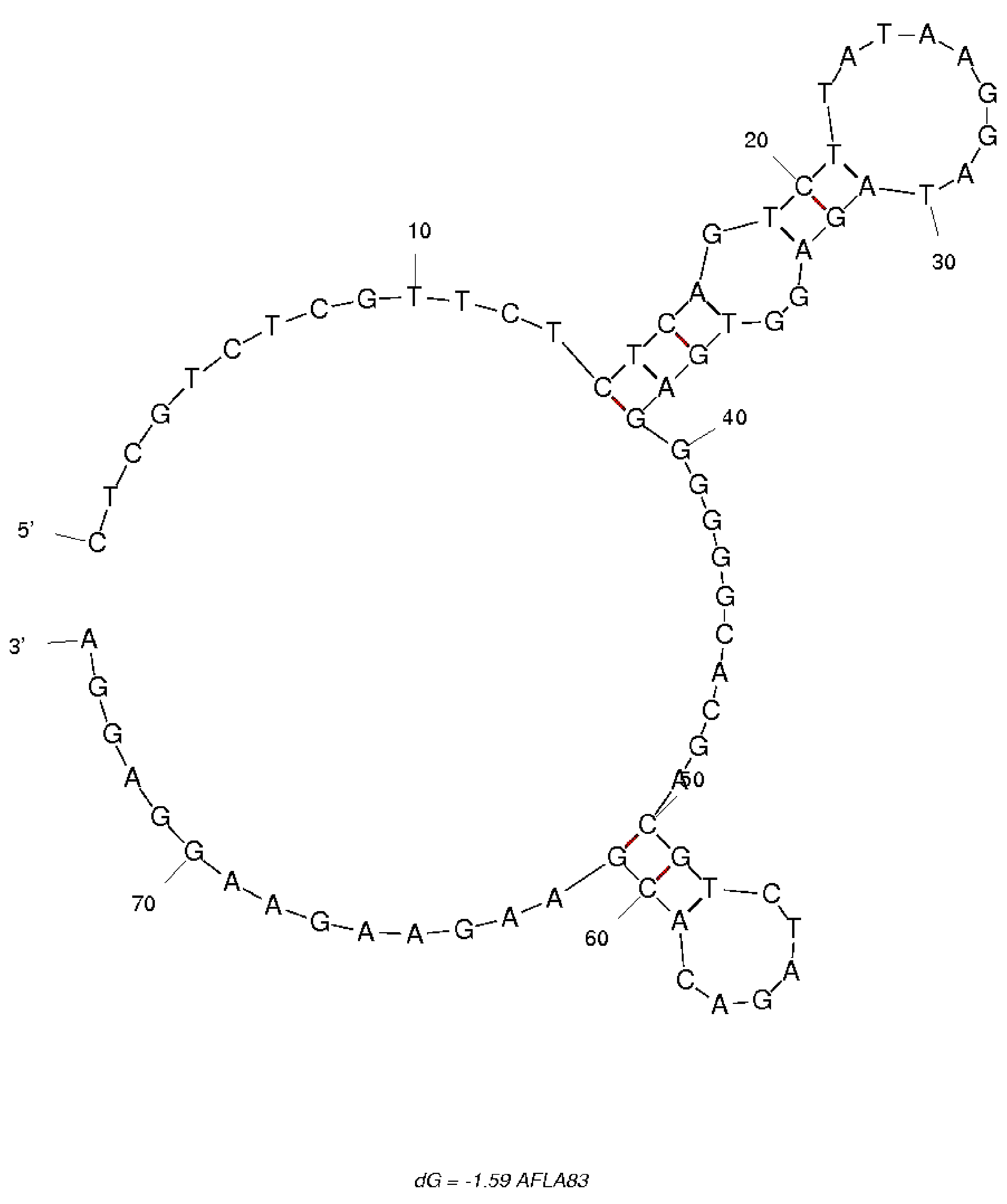


**AFLA83 (-1.59 kcal/mol)**


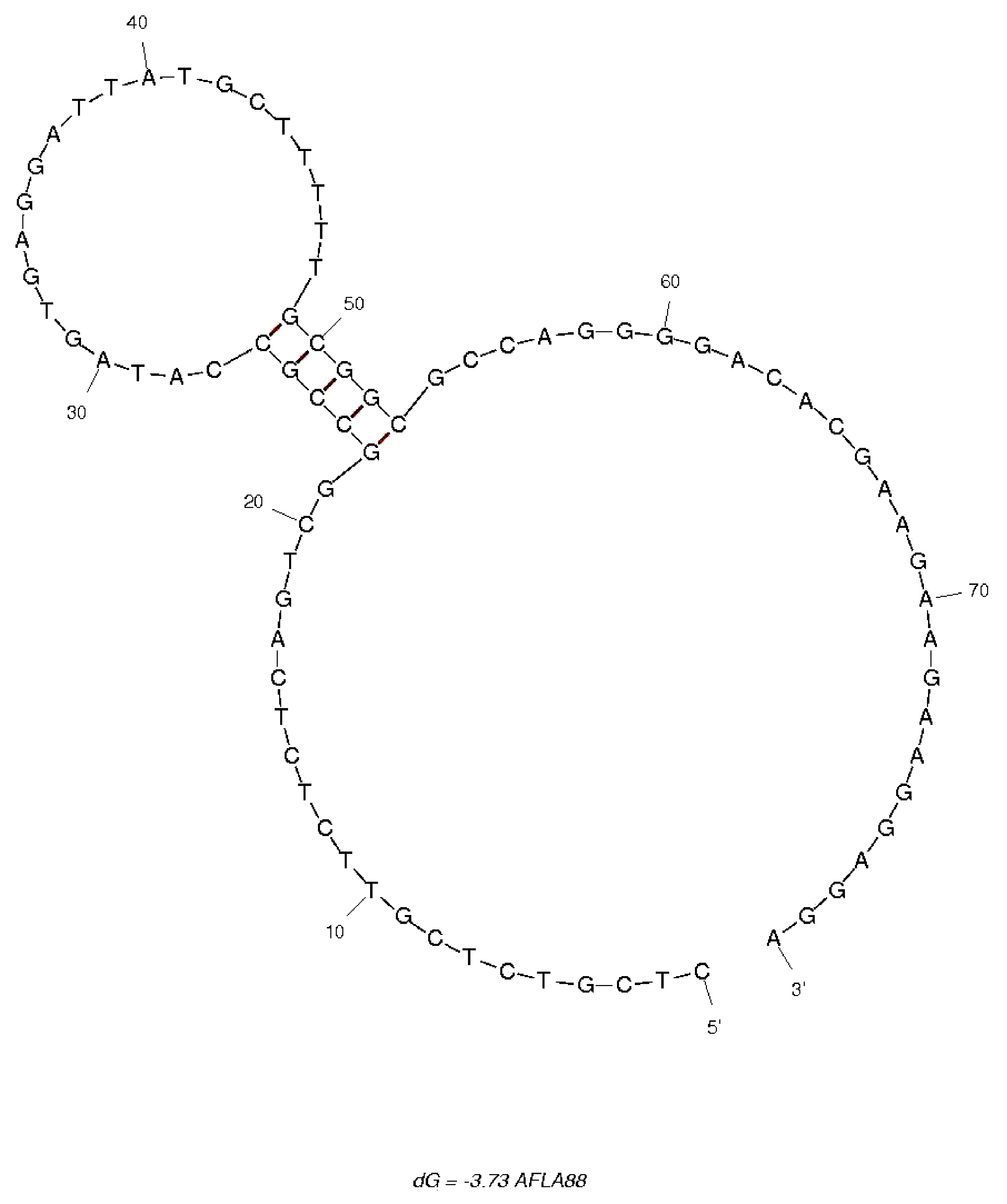


**AFLA88 (-3.75 kcal/mol)**

**Supporting figure.S4:**

Schematic illustration of ELONA for AFB1 determination: Immobilized AFB1+BSA (250 to 10 ng/ml) was incubated with anti - AFB1 biotinylated aptamer (250 nM) and streptavidin-HRP conjugate followed by subsequent enzyme label detection.


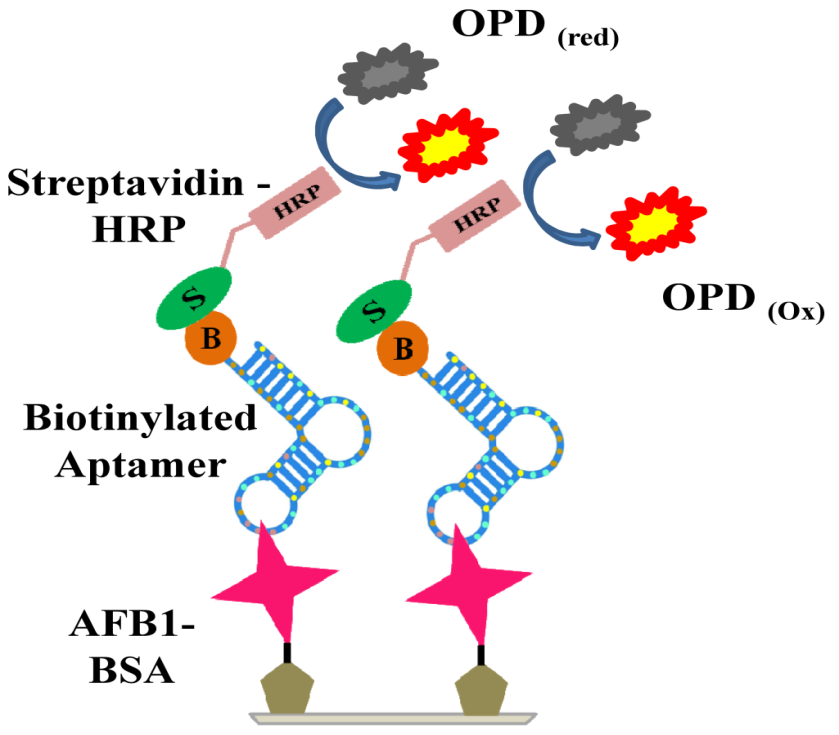


**Supporting figure.S5:**

Observation of ELONA, employing AFLA5, AFLA71 and AFLA53 in the detection of AFB1. The figure illustrates a significant gradation in the colour over background in each well from 250 to 10 ng/ml, and negligible colour development in negative control (0 ng/ml and BSA). The assay was performed in triplicates with individual aptamers.


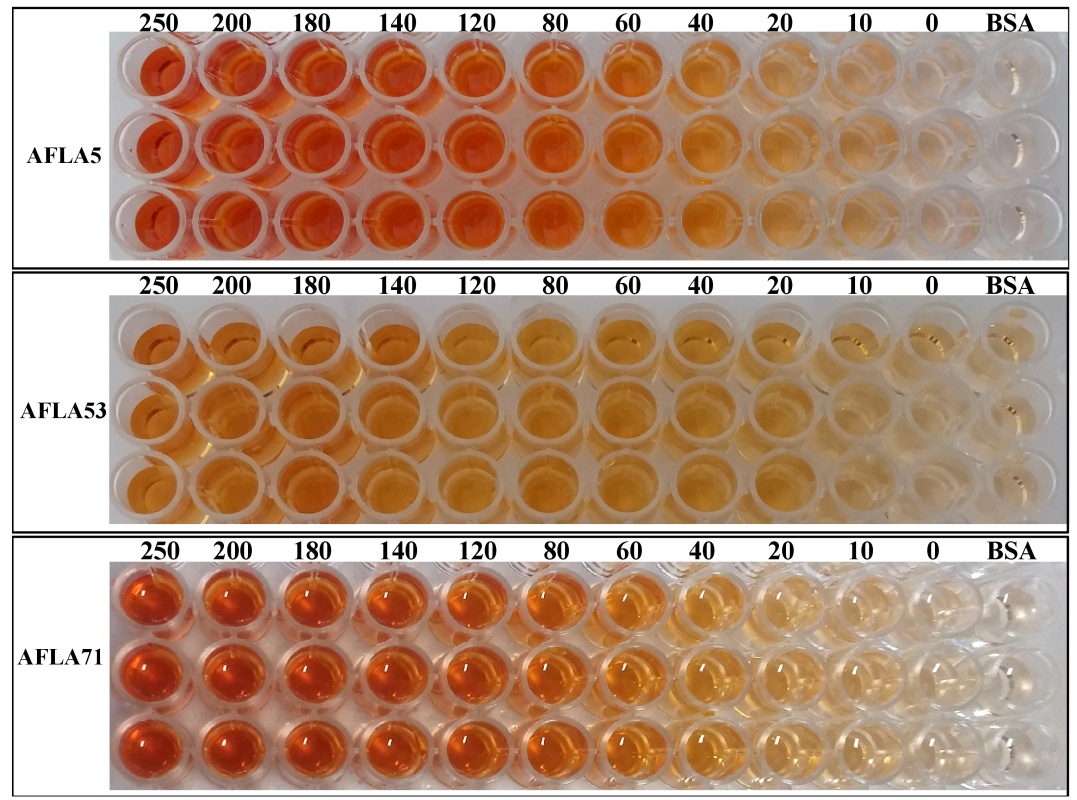

Supplement: Supplementary file 1 [file Data_Sheet_1.DOC]
